# Identification of a Master Regulator of Differentiation in *Toxoplasma*

**DOI:** 10.1016/j.cell.2019.12.013

**Published:** 2020-01-23

**Authors:** Benjamin S. Waldman, Dominic Schwarz, Marc H. Wadsworth, Jeroen P. Saeij, Alex K. Shalek, Sebastian Lourido

**Affiliations:** 1 Whitehead Institute for Biomedical Research, Cambridge, MA 02142, USA; 2 Department of Biology, Massachusetts Institute of Technology, Cambridge, MA 02142, USA; 3 Institute of Pharmacy and Molecular Biotechnology, University of Heidelberg, Im Neuenheimer Feld 364, 69120 Heidelberg, Germany; 4 Institute for Medical Engineering & Science, Department of Chemistry, and Koch Institute for Integrative Cancer Research, Massachusetts Institute of Technology, Cambridge, MA 02139, USA; 5 Broad Institute of MIT and Harvard, Cambridge, MA 02142, USA; 6 Ragon Institute of MGH, MIT and Harvard, Cambridge, MA 02319, USA; 7 Department of Pathology, Microbiology and Immunology, School of Veterinary Medicine, University of California, Davis, Davis, CA 95616, USA

**Keywords:** Toxoplasma gondii, differentiation, bradyzoite, chronic infection, master regulator, single-cell RNA-sequencing

## Abstract

*Toxoplasma gondii* chronically infects a quarter of the world’s population, and its recrudescence can cause life-threatening disease in immunocompromised individuals and recurrent ocular lesions in the immunocompetent. Acute-stage tachyzoites differentiate into chronic-stage bradyzoites, which form intracellular cysts resistant to immune clearance and existing therapies. The molecular basis of this differentiation is unknown, despite being efficiently triggered by stresses in culture. Through Cas9-mediated screening and single-cell profiling, we identify a Myb-like transcription factor (BFD1) necessary for differentiation in cell culture and in mice. BFD1 accumulates during stress and its synthetic expression is sufficient to drive differentiation. Consistent with its function as a transcription factor, BFD1 binds the promoters of many stage-specific genes and represents a counterpoint to the ApiAP2 factors that dominate our current view of parasite gene regulation. BFD1 provides a genetic switch to study and control *Toxoplasma* differentiation and will inform prevention and treatment of chronic infections.

## Highlights

• BFD1 is a master regulator of chronic-stage differentiation in *Toxoplasma gondii*
• Δ*BFD1* parasites fail to differentiate in cell culture or form cysts in infected mice
• Conditional expression of BFD1 is sufficient to induce differentiation
• BFD1 binds transcriptional start sites of genes induced during chronic stages

## Graphical Abstract

**Figure:**
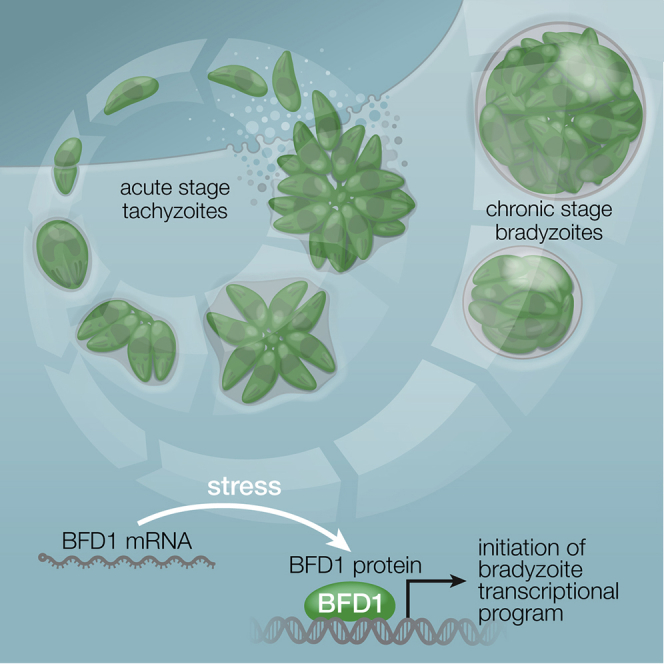

## Teaser

A single parasite transcription factor drives the differentiation of *Toxoplasma* to a cyst-forming stage to sustain chronic infection.

## Introduction

The duration of infection is a critical parameter in the evolutionary fitness of infectious organisms. Pathogens can extend the period of infection by establishing a latent or chronic state, avoiding clearance through slow replication, altered immunogenicity, and a diminished impact on the host. These reservoirs are frequently resistant to treatment due to decreased metabolic rates. Such persistent stages can recrudesce or contribute to transmission and are important barriers to curing and eradicating infectious diseases.

Chronic stages play important roles in the life cycles of many pathogens within the phylum Apicomplexa. *Plasmodium vivax* hypnozoites in the liver are resistant to many antimalarial therapies, leading to long periods of latency followed by the patent infection, complicating eradication efforts (Baird, 2009). *Toxoplasma gondii* tachyzoites are capable of invading any nucleated cell of warm-blooded animals, disseminating throughout the body and causing pathology through lysis of host cells. A proportion of tachyzoites differentiate into slow-growing bradyzoites, forming intracellular cysts with a tropism for brain and muscle tissue (Dubey et al., 1998). These cysts cannot be completely eliminated by the immune system or by current therapies, and, as a result, up to a quarter of the world’s population is chronically infected with *Toxoplasma* (Montoya and Liesenfeld, 2004). *Toxoplasma* infection is life-threatening in immunocompromised individuals, and a majority of these cases result from recrudescent infections (Porter and Sande, 1992). Approximately 2% of infections result in ocular lesions—a leading cause of infectious blindness—with high rates of reactivation from chronic stages that persist after treatment (Jones et al., 2015).

Major changes accompany the differentiation of rapidly proliferating tachyzoites into cyst-forming bradyzoites. The parasitophorous vacuole *Toxoplasma* replicates within is modified into a heavily glycosylated cyst wall, containing many stage-specific proteins of unknown function (Coppin et al., 2005, Ferguson, 2004, Tomita et al., 2013, Tomita et al., 2017, Tu et al., 2019). Parasite metabolism also changes drastically during differentiation, relying on anaerobic glycolysis instead of aerobic respiration and accumulating cytoplasmic starch granules (Denton et al., 1996, Guérardel et al., 2005, Sugi et al., 2017). Underpinning these dramatic changes in lifestyle, studies have identified hundreds to thousands of genes as differentially regulated between tachyzoites and bradyzoites (Behnke et al., 2008, Buchholz et al., 2011, Cleary et al., 2002, Manger et al., 1998, Pittman et al., 2014, Radke et al., 2005, Yahiaoui et al., 1999). While differentiation can be induced through a variety of methods in cell culture—alkaline pH, heat shock, small molecules, and nutrient starvation—the molecular mechanisms driving bradyzoite differentiation remain poorly understood (Fox et al., 2004, Radke et al., 2006, Soête et al., 1994).

While attempts to identify mutants unable to differentiate have yielded strains with decreased rates of stage conversion, linking these phenotypes to inactivation of individual genes has proved challenging (Matrajt et al., 2002, Singh et al., 2002). A single validated class of apicomplexan transcription factors, the AP2 DNA-binding proteins (ApiAP2s), has been extensively investigated as potential regulators of differentiation. Knockouts of individual ApiAP2s modulate, but ultimately fail to completely ablate, bradyzoite differentiation, leading to the model that no master transcriptional regulator of this process exists in *Toxoplasma* (Jeffers et al., 2018). Overturning this view, we describe the identification and characterization of a single transcription factor, Bradyzoite-Formation Deficient 1 (BFD1), which is both necessary and sufficient for differentiation in cell culture and during mouse infection. This discovery provides a unique molecular handle to study the chronic stages of infection, which represents a major barrier for developing live-attenuated vaccines and radical cures against *Toxoplasma*.

## Results

### Generation of a Differentiation Reporter Compatible with Cas9-Mediated Screens

**Figure 1:**
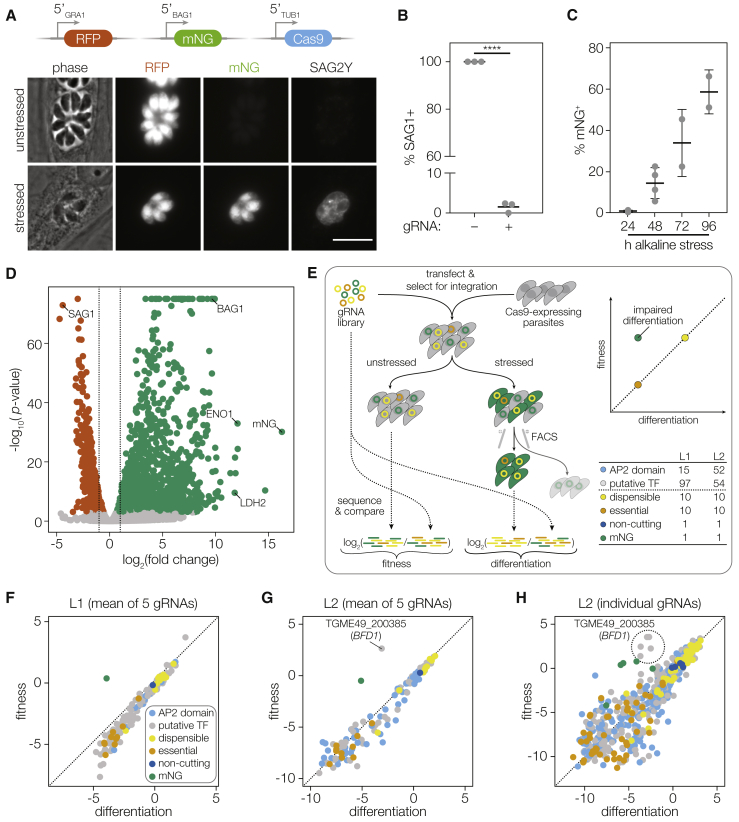
A Genetic Screen Identifies a Putative Regulator of *T. gondii* Differentiation (A) Construction of a differentiation reporter strain that constitutively expresses RFP and Cas9 and conditionally expresses mNeonGreen (mNG) under the regulation of the bradyzoite-specific *BAG1* promoter. SAG2Y is a bradyzoite-specific surface marker. Images were taken after 48 h of growth under unstressed or alkaline-stressed conditions. Scale bar is 10 μm. (B) Transfection and selection for a gRNA targeting the surface antigen SAG1 resulted in gene disruption in 98% of the resulting population. The mean was plotted for n = 3 biological replicates; 92–102 vacuoles were scored for each replicate; ^∗∗∗∗^p < 0.0001 by Student’s two-tailed t test. (C) Percentage of alkaline-stressed reporter parasites expressing mNG, quantified by FACS. The mean ± SD was plotted for n = 2–4 biological replicates. (D) RNA sequencing and differential expression (DE) analysis identified 1,311 genes as significantly upregulated (green) and 933 genes as significantly downregulated (red) in bradyzoites (adjusted p < 0.001), with 1,240 and 700 changing 2-fold or more, respectively (dotted lines). The analysis was based on n = 3 independent experiments. (E) Screening and analysis workflow. The log_2_-transformed fold changes from the input library to the final unstressed or stressed mNG^+^ populations are defined as fitness or differentiation scores, respectively. (F and G) Fitness and differentiation scores at the gene level following screening L1 (F) or L2 (G). (H) Fitness and differentiation scores for individual gRNAs in L2. See also Figure S1 and Data S1 and S2.

**Figure S1:**
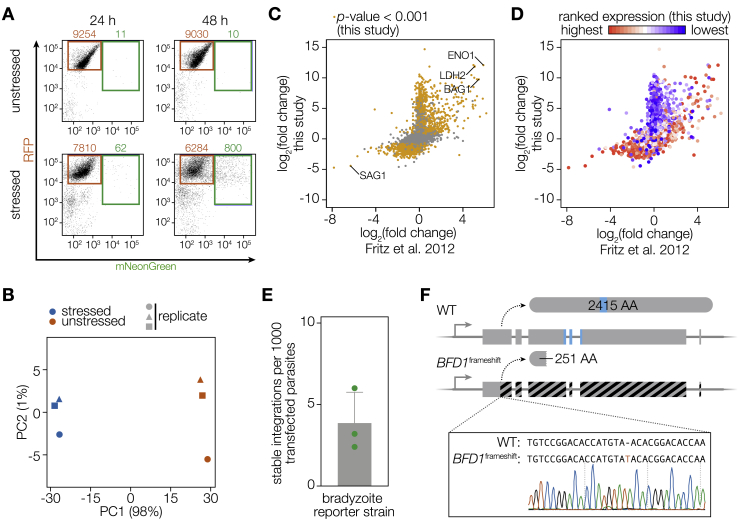
Stage-Specific RNA-Seq and Forward-Genetic Screening in a Differentiation Reporter Strain, Related to Figure 1 (A) Sample FACS plots for the bradyzoite reporter strain at 24 or 48 h of growth under unstressed or stressed conditions. 10,000 events per plot. Gates for mNG^+^ and mNG^–^ populations are highlighted. (B) Principal component analysis of stage-specific RNA-seq replicates. (C**–**D) Comparison of differentially expressed genes identified in this study and a previously published dataset (Fritz et al., 2012). Color assigned by adjusted *p-value* < 0.001 (C) or rank of base mean expression as calculated by DESeq2 (D). (E) Efficiency of construct integration into the bradyzoite reporter strain. Plaquing efficiency was compared between parasites transfected and selected for integration of a plasmid encoding a gRNA against *SAG1* and the pre-transfection population to obtain a viability-normalized integration rate. Mean ± SD is plotted for n = 3 independent experiments. (F) Generation of a *BFD1*-deficient reporter strain by introducing a frameshift using Cas9. The bradyzoite reporter strain was transfected with a plasmid encoding a gRNA targeting the first exon of TGME49_200385 (*BFD1*) to isolate a strain with a frameshift mutation (*BFD1*^frameshift^), resulting in a premature stop codon after amino acid 251.

To screen for mutants deficient in differentiation, we modified the ME49 strain of *Toxoplasma* to be compatible with Cas9-mediated gene disruption and enrichment of differentiated parasites (Sidik et al., 2014, Sidik et al., 2016). Our reporter strain constitutively expresses RFP and conditionally expresses the bright green fluorescent protein mNeonGreen (mNG) under the promoter of the canonical bradyzoite-specific gene *BAG1* (Figure 1A). Our reporter strain further expresses Cas9 to permit efficient gene inactivation, as shown by disruption of the major tachyzoite surface antigen *SAG1* in 98% parasites transfected with a guide RNA (gRNA) targeting the locus (Figure 1B). Growth of the reporter strain under alkaline stress, which induces *Toxoplasma* differentiation in cell culture, resulted in increasing proportions of parasites expressing mNG (Figure 1C; Figure S1A).

To characterize transcriptomic differences between tachyzoites and bradyzoites, we performed stage-specific bulk RNA sequencing (RNA-seq) using our reporter strain. We compared gene expression of fluorescence-activated cell sorting (FACS)-purified tachyzoites (mNG^–^, 24 h unstressed growth) to bradyzoites (mNG^+^, 48 h stressed growth) and identified 1,311 genes as upregulated and 933 genes as downregulated in bradyzoites (Figure 1D; Data S1; STAR Methods). This comparison was chosen based on reproducible differences observed between 24 and 48 h unstressed replicates, which likely represent increased numbers of extracellular parasites following the completion of the lytic cycle at 48 h. Principal component analysis showed that 98% of variance is explained by growth condition, with minimal batch effects (Figure S1B). Highly regulated genes agreed with previous datasets, with the canonical bradyzoite-specific genes *BAG1*, *LDH2*, and *ENO1* strongly upregulated, and the major tachyzoite surface antigen *SAG1* strongly downregulated (Figure 1D; Figure S1C). Genes that were not previously annotated as differentially regulated tended to have lower expression, suggesting enhanced sensitivity in our dataset (Figure S1D).

### Genetic Screening Identifies a Putative Regulator of *T. gondii* Differentiation

Modeled on our previous CRISPR-based screens in the lab-adapted RH strain of *Toxoplasma* (Sidik et al., 2016), we developed a screen for differentiation in a strain that retains normal stage conversion (ME49). However, the lower viability and integration rates we observed in this background—approximately 100 times lower than previously measured for RH—limited the number of genes that could be screened simultaneously to the low hundreds (Figure S1E). By combining differential expression analysis with domain annotation and gene ontology, we assembled two libraries, each targeting ∼100 potential nucleic acid–binding proteins, with 5 gRNAs per gene. Library 1 (L1) largely consisted of genes identified as differentially regulated in our preliminary RNA-seq experiment, while Library 2 (L2) contained genes with DNA-binding domains commonly found in transcription factors, such as zinc finger and Myb-like domains. Across both libraries, all 67 members of the ApiAP2 transcription factor family were targeted, along with 151 putative nucleic acid-binding proteins (Figure 1E; Data S2) (Balaji et al., 2005, Painter et al., 2011). As controls, each library additionally targeted 10 genes known to be essential and 10 genes known to be dispensable, and contained 10 non-cutting gRNAs and 5 gRNAs against the mNG reporter itself.

Following transfection of the libraries, parasites were maintained in selective medium for four passages to allow for integration of the gRNA plasmids and inactivation of the targeted genes, before splitting the population between unstressed or alkaline-stressed conditions. After 10 days, bradyzoites (mNG^+^ parasites) from the stressed populations were isolated by FACS. Integrated gRNAs from the tachyzoites (final unstressed population) or from the bradyzoites (mNG^+^ stressed population) were amplified and sequenced along with the input library. The fold change in relative abundance was calculated per gRNA between each final sample and the library. The mean log_2_ fold change for guides against each gene are referred to as fitness or differentiation scores, based on comparisons to the unstressed or bradyzoite samples, respectively. Candidate genes should be depleted specifically in the bradyzoite population (low differentiation score relative to their fitness score), as should gRNAs against the mNG reporter. In L1, only control mNG gRNAs had low differentiation scores compared to their fitness scores (Figure 1F). In L2, however, gRNAs targeting a single gene—TGME49_200385, which we name Bradyzoite-Formation Deficient 1 (*BFD1*)—were specifically depleted in the differentiated populations along with control mNG gRNAs (Figures 1G and 1H). Failure of a *BFD1* mutant to express the mNG reporter following alkaline stress was confirmed by transfecting a single *BFD1* targeting gRNA into our reporter strain. A frameshifted clone was isolated with a single nucleotide insertion at the cut site (*BFD1*^frameshift^, Figure S1F). Under alkaline stress, the wild-type (WT) reporter strain showed robust mNG expression, while the *BFD1*^frameshift^ clone did not (Video S1).

Video S1. The *BFD1*^frameshift^ Reporter Strain Fails to Express mNG under Alkaline Stress, Related to Figure 1 Parasites were allowed to invade host cells for 4 h under unstressed conditions, then shifted to alkaline stressed conditions. One image was taken per h over 72 h. RFP is in magenta and mNG is in green. Scale bar is 20 μm.

### BFD1 Is Homologous to c-Myb and Conserved among Cyst-Forming Coccidians

**Figure S2:**
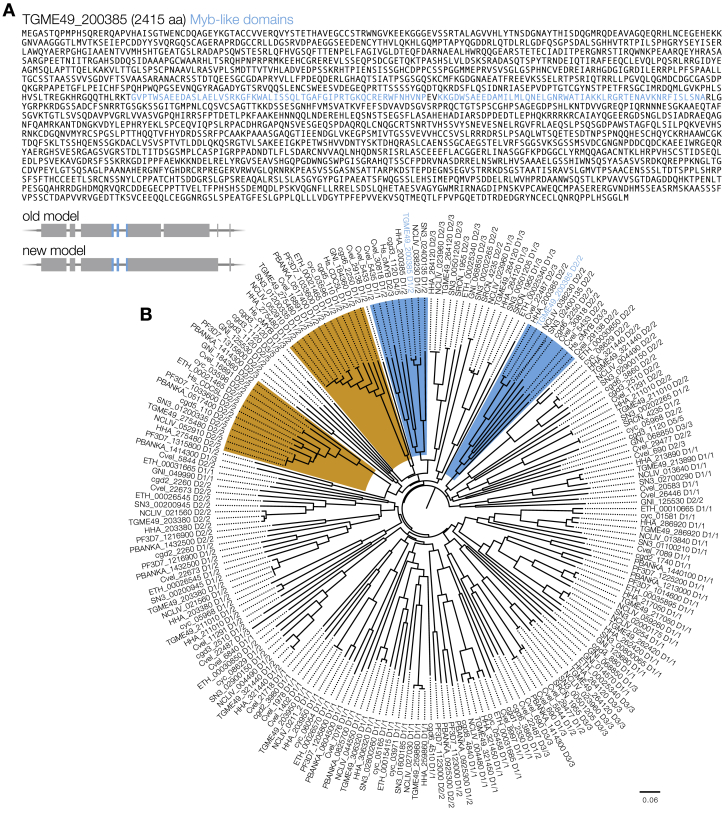
An Updated *BFD1* Gene Model and Phylogeny, Related to Figure 2 (A) Updated gene model and protein sequence of TGME49_200385. Sequencing of cDNA confirmed changes to the gene model after the 5^th^ exon annotated on ToxoDB v. 42, which change the reading frame of the latter third of the protein. DNA-binding domains (SM00717) highlighted in blue. (B) Neighbor-joining phylogenetic tree of SANT/Myb-like DNA-binding domains (SM00717) present in representative apicomplexan genomes, along with human c-Myb and CDC5L. Clades containing c-Myb and CDC5L are highlighted in blue and orange, respectively. Alignment performed using ClustalW. Scale bar is substitutions per site.

**Figure 2:**
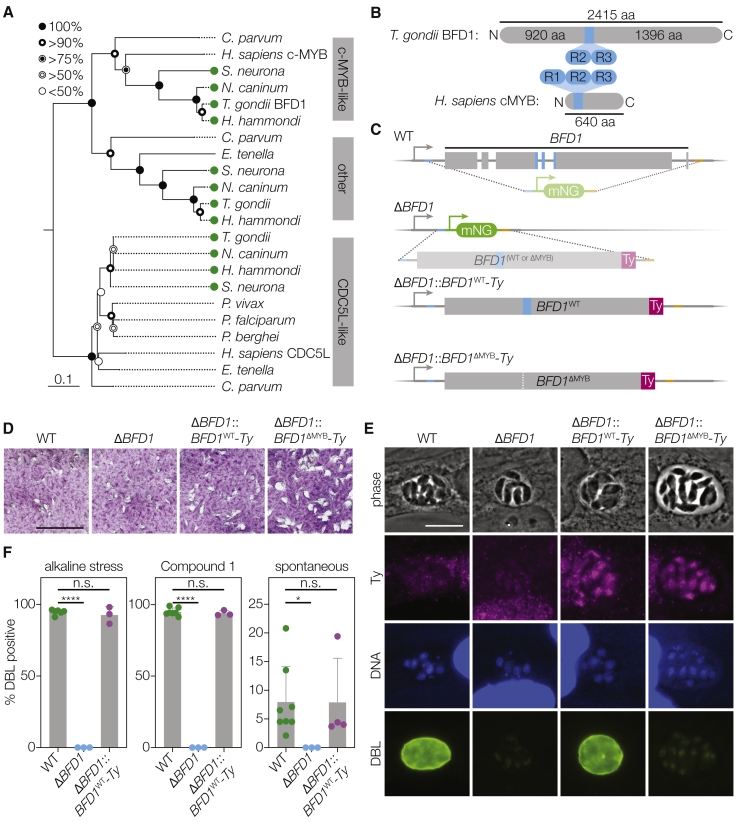
BFD1 Is a Nuclear Factor Necessary for Differentiation in Cell Culture (A) Neighbor-joining tree showing the phylogenetic relationship of the concatenated Myb-like domains from BFD1 and its closest homologs in other apicomplexans and humans. Tissue-cyst-forming species are indicated (green circles). Bootstrap values for 1,000 trials are displayed. Scale bar is substitutions per site. (B) Diagram of BFD1 and human c-Myb highlighting the Myb-like domains (blue). The DNA-binding repeats of BFD1 are similar to the second and third repeats of c-Myb. (C) Generation of Δ*BFD1* and *BFD1*^WT^ or *BFD1*^ΔMYB^ complemented parasites. To create Δ*BFD1* parasites, the endogenous coding sequence was replaced with a fluorescent cassette. The knockout was complemented with a wild-type (WT) (*BFD1*^WT^) or DNA-binding deficient (*BFD1*^ΔMYB^) Ty-tagged allele at the endogenous locus. (D) Plaque assays of indicated strains grown under unstressed conditions for 14 days. Scale bar is 1 cm. (E) Representative vacuoles after 48 h of alkaline stress. FITC-labeled *Dolichos biflorus* lectin (DBL) specifically stains differentiated vacuoles. Ty was stained with BB2 (magenta), and DNA was stained with Hoechst (blue). Scale bar is 10 μm. (F) Quantification of differentiation in WT, knockout, and complemented parasites following 48 h of alkaline stress, 48 h of compound 1 treatment, or occurring spontaneously under unstressed conditions in the same time frame. The mean ± SD was plotted for n = 3–8 biological replicates, with percentage of DBL positive vacuoles calculated from at least 100 vacuoles per replicate. ^∗∗∗∗^p < 0.0001, ^∗^p < 0.05, Student’s one-tailed t test. See also Figure S2.

We defined the sequence of the *BFD1* open reading frame based on cDNA sequencing, which differed from the annotated gene model and encoded a protein of 2,415 amino acids (Figure S2A). BFD1 contains two tandem SANT/Myb-like DNA-binding domains (SMART accession 00717), flanked by large extensions lacking identifiable motifs. Phylogenetic analysis of the DNA-binding domains shows that BFD1 and syntenic orthologs from other tissue-cyst-forming species comprise a clade with human c-Myb, distinct from CDC5L-like sequences, with their first and second domains displaying homology to the R2 and R3 repeats of the prototypical c-Myb, respectively (Figures 2A and 2B; Figure S2B).

### Loss of BFD1 Blocks Parasite Differentiation Regardless of Induction Method

To provide a clean background for precise genetic manipulation, we generated a low-passage, non-homologous end joining (NHEJ)-deficient ME49 strain through deletion of *KU80* (STAR Methods). In this background, we replaced the entire coding sequence of *BFD1* with an mNG expression cassette, creating a Δ*BFD1* strain (Figure 2C). We then complemented Δ*BFD1* parasites by introducing Ty-tagged cDNA copies of *BFD1* at the endogenous locus, either full-length (Δ*BFD1*::*BFD1*^WT^*-Ty*) or with its Myb-like domain deleted (Δ*BFD1*::*BFD1*^ΔMYB^*-Ty*). Deletion of *BFD1* and subsequent complementation caused no defect in tachyzoite growth as assayed by plaque formation. The complemented strains grew slightly faster, perhaps due to prolonged passaging in cell culture (Figure 2D). Differentiating vacuoles can be identified using *Dolichos biflorus* lectin (DBL) staining, which recognizes *N*-acetylgalactosamine on the bradyzoite-specific cyst-wall protein CST1 (Tomita et al., 2013). Many cyst wall proteins, including CST1, are robustly detected in large proportions of vacuoles after only 24 h under alkaline stress, making DBL positivity an earlier and more robust marker for differentiating parasites than *BAG1* transcription which accumulates more slowly (Figure 1C). WT vacuoles became robustly DBL^+^ after 48 h under alkaline stress. By contrast, no Δ*BFD1* vacuoles developed DBL positivity under identical conditions. Complementation with the WT but not the ΔMYB allele of *BFD1* restored differentiation and revealed nuclear localization of the transgenes (Figures 2E and 2F). Δ*BFD1* parasites also failed to differentiate spontaneously or when induced with the small molecule Compound 1 (Figure 2F), and in both cases complementation with full-length *BFD1* restored differentiation to WT levels (Radke et al., 2006).

### Δ*BFD1* Parasites Fail to Form Brain Cysts in Mice

**Figure 3:**
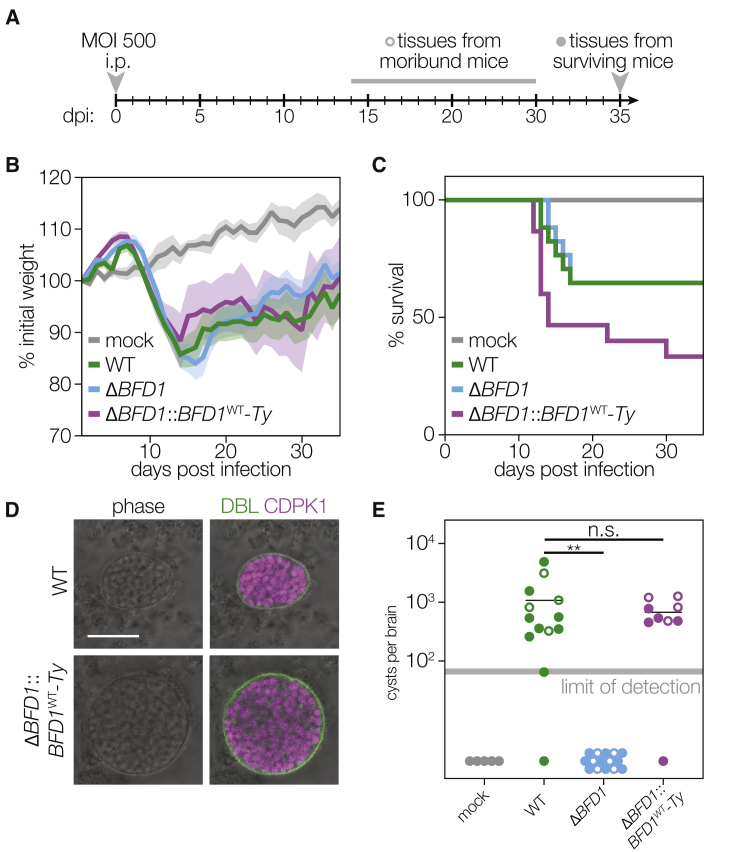
BFD1 Is Necessary for Formation of Brain Cysts in Mice (A) Timeline of mouse infections. Groups of CD-1 female mice were inoculated i.p. with 500 tachyzoites per animal from each strain or mock inoculated with PBS. Cyst formation was assayed in moribund animals starting 2 weeks post-infection and in all surviving animals at 5 weeks post-infection. (B) Mean normalized weights of animals in each group. Graph represents mean ± SEM for all surviving animals at a given time point. Graphs are for n = 5 mock-inoculated mice and n = 15 for each parasite strain. (C) Survival curve of animals in (B). (D) Representative cysts from WT and Δ*BFD1*::*BFD1*^WT^-infected animals. The cyst wall was stained with DBL (green) and individual parasites with anti-CDPK1 (magenta). Scale bar is 20 μm. (E) Cyst burden per animal, denoting those sacrificed before (open circles) or after (closed circles) 5 weeks of infection. Cysts per brain were estimated from counting four blinded replicates, with a limit of detection of 56–71 cysts per brain, depending on the volume of the sample analyzed. Mean is plotted for each group. ^∗∗^p < 0.01, Student’s one-tailed t test. See also Figure S3.

**Figure S3:**
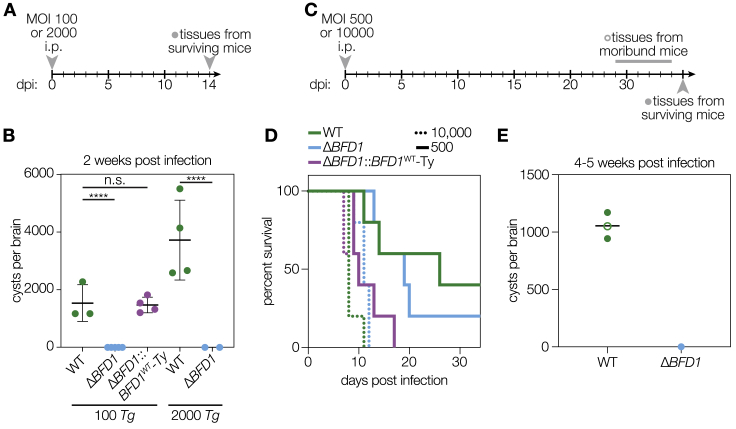
Virulence and Brain Cyst Formation by Δ*BFD1* Parasites in CBA/J Mice, Related to Figure 3 (A**–**B) In groups of 5, female CBA/J mice were inoculated with 100 or 2,000 tachyzoites i.p. of WT, Δ*BFD1*, or Δ*BFD1*::*BFD1*^WT^ and surviving animals were sacrificed 2 weeks post-infection to assay brain cyst formation (A). Cyst burdens were estimated by counting 4 blinded samples from each animal. Mean ± SD is plotted with each dot representing an animal; ^∗∗∗∗^p < 0.0001, Student’s one-tailed t test (B). (C**–**E) In groups of 5, female CBA/J mice were inoculated with 500 or 10,000 tachyzoites i.p of WT, Δ*BFD1*, or Δ*BFD1*::*BFD1*^WT^. Starting at 3 weeks post-infection, brains were isolated from moribund animals, and at 5 weeks post-infection all surviving animals were sacrificed (C). Survival curve of animals infected with 10,000 (dotted lines) or 500 (solid lines) tachyzoites (D). Brain cyst burden of moribund or sacrificed animals, estimated by counting 4 blinded samples from each animal. Mean ± SD is plotted with each dot representing an animal (E).

To determine whether BFD1 is necessary for the formation of tissue cysts in animals, we assessed acute virulence and chronic infection of mice. CD-1 female mice were infected by intraperitoneal injection with 500 tachyzoites of WT, Δ*BFD1*, or Δ*BFD1*::*BFD1*^WT^ parasites (Figure 3A). Morbidity and mortality were comparable among the three strains (Figures 3B and 3C), indicating that *BFD1* is dispensable for the acute symptoms of *Toxoplasma* infection. The marginal increase in Δ*BFD1*::*BFD1*^WT^ virulence may be attributed to the faster growth rate observed by plaque formation (Figure 2D). Brain cysts from WT and Δ*BFD1*::*BFD1*^WT^ infections were morphologically identical, with a DBL positive cyst wall surrounding hundreds of bradyzoites (Figure 3D). Starting at 2 weeks post-infection, cyst burden was measured by examining the brains of infected animals. Cyst numbers ranged from several hundred to several thousand per animal infected with WT or *BFD1*::*BFD1*^WT^ parasites; however, cysts were never isolated from Δ*BFD1*-infected animals (Figure 3E). Similar results were observed in experiments conducted with CBA/J mice (Figure S3). Taken together, these results reveal that loss of *BFD1* results in a specific and complete defect in tissue cyst formation during animal infections.

### Characterizing Differentiation by Single-Cell RNA Sequencing

**Figure S4:**
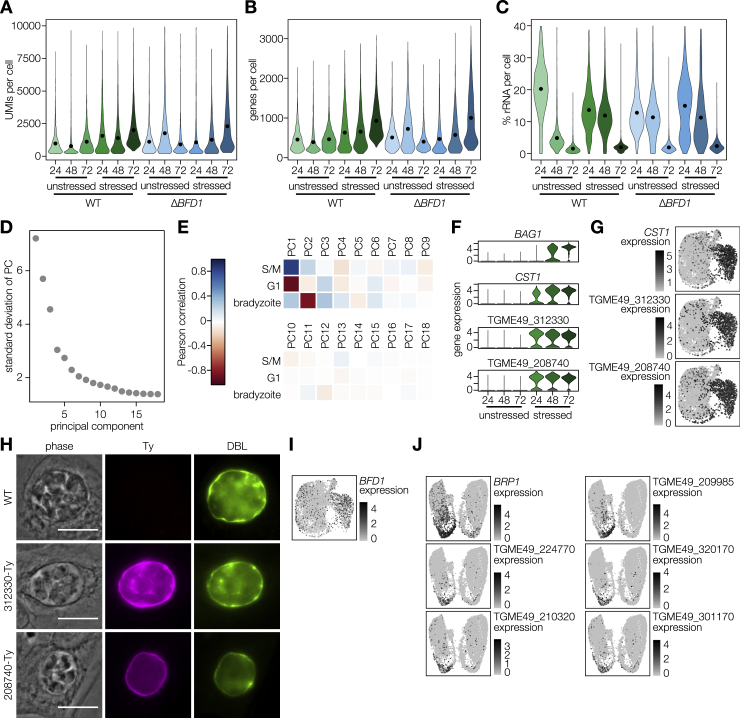
Profiling *Toxoplasma* Differentiation at Single-Cell Resolution, Related to Figure 4 (A**–**B) Distribution of UMIs (A) or unique genes detected (B) across single cells from indicated samples and time points. Pre-processing quality control cutoffs required a minimum of 200 and a maximum of 10,000 UMIs. (C) Percentage of UMIs corresponding to ribosomal genes. Pre-processing quality control cutoffs allowed a maximum of 40% rRNA reads. (D) The majority of variance between cells is driven by cell-cycle and stage-specific genes. Plotting the 18 principal components (PCs) determined to be statistically significant by permutation analysis. The first three PCs explain 66.4% of variance at the 72 h time point. (E) Pearson correlations of cell embeddings in PCs 1–18 to cell scores for G1, S/M, or bradyzoite-specific gene signatures. (F) Violin plots of expression of early bradyzoite marker genes in wild-type parasites after 24, 48 or 72 h of growth under unstressed or stressed conditions. (G) UMAP visualization as in Figure 4E colored by expression of *CST1* or other early bradyzoite markers. (H) Endogenous tagging of two early bradyzoite markers shows localization to the cyst wall. Cultures were fixed and stained after 72 h under alkaline stress. Scale bar is 10 μm. (I) *BFD1* is not stage-specific but is modestly overrepresented in bradyzoite-containing clusters. UMAP visualization as in Figure 4E colored by expression of *BFD1*. (J) UMAP visualizations colored by expression of marker genes identified as specifically upregulated in bradyzoites during the S/M phase of the cell cycle.

**Figure 4:**
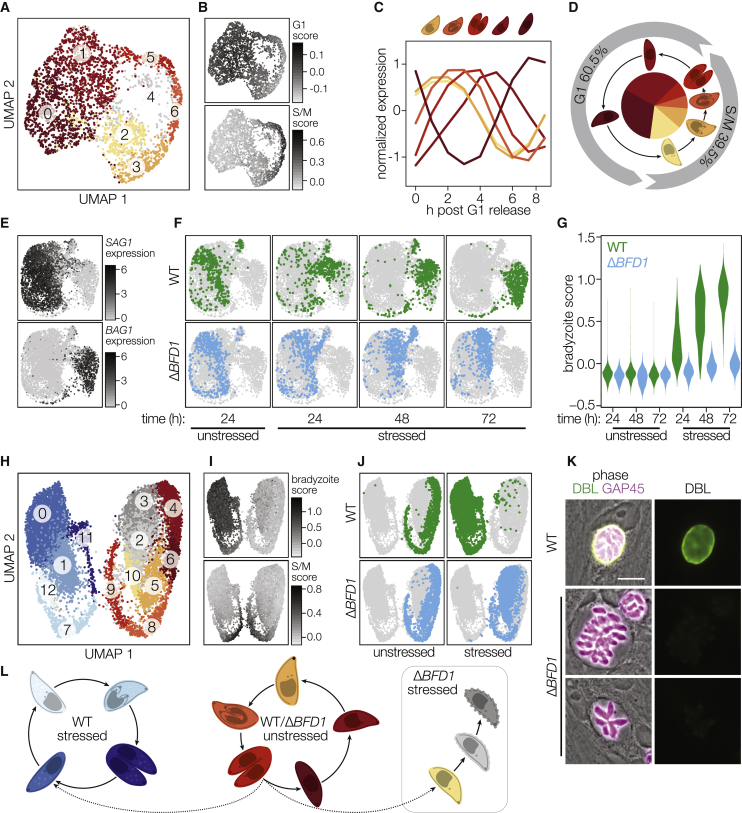
BFD1 Is Required to Express Bradyzoite Genes and Initiate Differentiation (A) Clustering of unstressed parasites of both genotypes after 72 h of growth (3,149 cells total) visualized by uniform manifold approximation and projection (UMAP). (B) UMAP from (A) shaded by score for expression of known G1 or S/M-specific gene sets. (C) Average expression profiles of all genes differentially expressed within each cluster identified in (A) across a microarray dataset of synchronized tachyzoites (Behnke et al., 2010). Colors correspond to those used in (A). (D) Proportion of cells in G1 (0 and 1) or S/M (2, 3, 5, and 6) clusters. (E) Cells shaded by expression of the canonical stage-specific genes *SAG1* and *BAG1* following clustering of all parasites from all time points, genotypes, and growth conditions. UMAP visualization is downsampled to 500 cells from each combination of time point, genotype, and growth condition (6,000 cells total). (F) UMAP as in (E), with cells highlighted by sample of origin for WT (green) or Δ*BFD1* (blue) parasites. (G) Distribution of cell scores for the expression of genes highly upregulated in bradyzoites. (H) UMAP of all WT and Δ*BFD1* parasites from unstressed and stressed cultures at 72 h time point. (I and J) UMAP projection from (H), shaded by scores for expression of highly upregulated bradyzoite genes as in G, or S/M-specific genes as in (B) (I) or colored by sample of origin (J). (K) Representative WT and Δ*BFD1* vacuoles at 72 h post-alkaline stress. GAP45 is a marker for the inner membrane complex. Scale bar is 10 μm. (L) Proposed model of cell-cycle progression for WT and Δ*BFD1* parasites under the different treatments. See also Figure S4 and Data S3.

To profile the asynchronous process of differentiation and determine the extent of defects exhibited by Δ*BFD1* parasites, we performed single-cell RNA sequencing (scRNA-seq) of *T. gondii* using Seq-Well (Gierahn et al., 2017, Hughes et al., 2019). WT or Δ*BFD1* parasites were grown under unstressed or stressed conditions (24, 48, or 72 h), mechanically released from host cells, and analyzed. Following downstream processing and alignment, 26,560 cells passed quality-control cutoffs with, on average, 1,537 unique molecular identifiers (UMIs, a proxy for unique transcripts) and 685 genes represented per cell (Figures S4A–S4C; STAR Methods). As cells from the 72 h time point were of the highest quality, we clustered unstressed parasites from this time point to examine the tachyzoite cell cycle. Seven clusters were identified, with six arranged in a circular pattern in a uniform manifold approximation and projection (UMAP) visualization (Figure 4A) (McInnes et al., 2018). Scoring cells based on expression of known cell-cycle signatures identified clusters 0 and 1 as G1-like and clusters 2, 3, 5, and 6 as S/M-like (Figure 4B) (Behnke et al., 2010, Suvorova et al., 2013). Cluster 4 lacks strong G1 or S/M identity and any uniquely defining genes; it was therefore disregarded in subsequent analyses. We identified 1,173 genes as upregulated in at least one cluster compared to all the others, including 525 genes not previously identified as cell-cycle regulated. Plotting the average expression profile of these markers across an existing dataset of synchronized tachyzoite gene expression revealed a progression through the cell cycle in a counterclockwise direction around the plot (Figures 4A and 4C; Data S3) (Behnke et al., 2010). The proportion of cells identified as being in G1 or S/M matches the 60:40 ratio previously measured for these stages (Figure 4D) (Radke et al., 2001).

Clustering cells from all time points, growth conditions, and genotypes revealed a clear division between tachyzoite (SAG1^+^) and bradyzoite (BAG1^+^) clusters (Figure 4E). Analysis of the significant principal components underlying this clustering suggested the majority of variance between cells is attributable either to the cell-cycle or differentiation state (Figures S4D and S4E). Under alkaline stress, WT parasites quickly exit the tachyzoite cell cycle and begin progressing toward bradyzoite-containing clusters (Figure 4F). Scoring cells based on their expression of 99 genes identified consistently as upregulated in bradyzoites across four independent datasets (Behnke et al., 2008, Fritz et al., 2012, Ramakrishnan et al., 2019) shows a clear increase in this signature over time (Figure 4G; Data S6). In agreement with the robust DBL positivity seen at 48 h post-induction, *CST1* is among the earliest genes upregulated during differentiation (Figures S4F and S4G; Data S3). Endogenous tagging of two additional early markers of differentiation (TGME49_312330, TGME49_208740) identified both as cyst-wall proteins and confirmed the localization recently shown for TGME49_208740 (Figure S4H) (Tu et al., 2019). Within this scRNA-seq data, *BFD1* is only modestly upregulated at 72 h in stressed WT parasites (Figure S4I; Data S3). UMAP visualization of unstressed or stressed parasites at the 72 h time point showed two circular patterns, driven primarily by cell cycle, and bradyzoite-specific gene signatures (Figures 4H and 4I). We identified 228 genes as upregulated specifically in replicating bradyzoites (clusters 7, 11, and 12), including the previously identified Bradyzoite Rhoptry Protein 1 (Figure S4J; Data S3) (Schwarz et al., 2005). In WT parasites, 19% of bradyzoites are in clusters expressing S/M markers, reflecting their replication at roughly half the rate observed for tachyzoites (Dzierszinski et al., 2004).

### BFD1 Is Necessary for Bradyzoite Differentiation

Under unstressed conditions, the distribution of Δ*BFD1* parasites at 72 h mirrors that of WT parasites along the various tachyzoite clusters. However, tracking the fate of Δ*BFD1* parasites under stress conditions demonstrates that they continue to replicate as tachyzoites until the 72 h time point, when they cluster separately from both WT tachyzoites and bradyzoites (Figure 4F). Scoring these cells based on their expression of bradyzoite-specific genes revealed a widespread failure of Δ*BFD1* parasites to initiate bradyzoite-specific gene expression (Figure 4G; Data S3). Under alkaline stress, 7.2% of Δ*BFD1* parasites clustered with dividing S/M phase tachyzoites, while 88.6% organized into three G1-adjacent clusters (2, 3, and 10) distinct from those harboring G1 tachyzoites (Figure 4J). Aberrant morphologies observed for Δ*BFD1* parasites after 72 h under stress suggest that these G1-adjacent clusters contain dying parasites, although we cannot exclude the existence of a G1-arrested state (Figure 4K). We propose that, due to the necessity of *BFD1* for differentiation, after 72 h under alkaline stress, Δ*BFD1* parasites are dying due to their failure to respond appropriately to that stress (Figure 4L).

### BFD1 Is Sufficient to Drive Bradyzoite Differentiation

**Figure 5:**
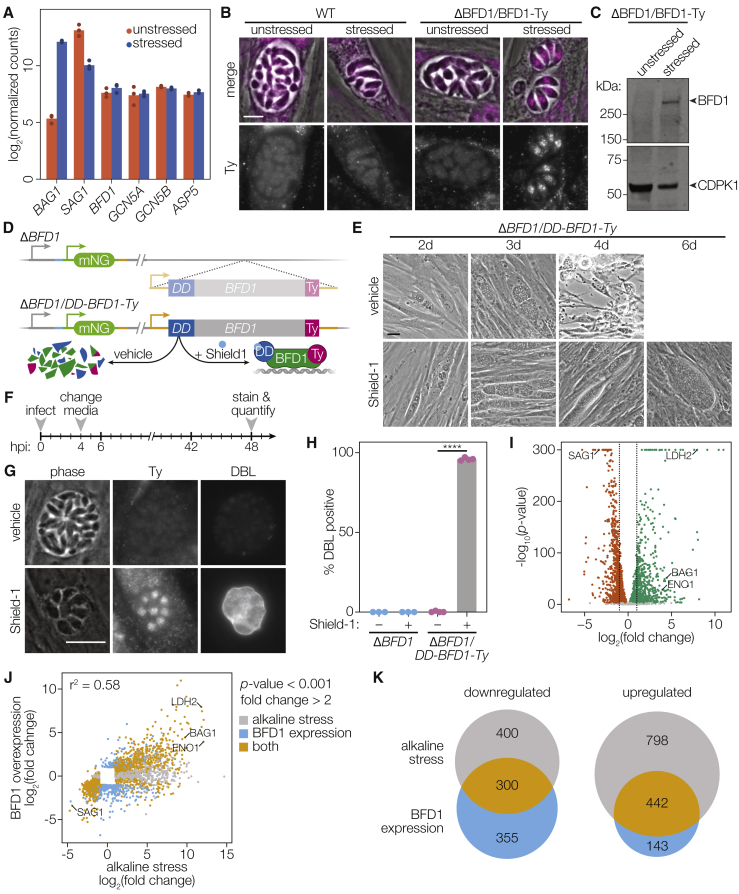
BFD1 Is Sufficient to Drive Differentiation in the Absence of Stress (A) Normalized counts for selected genes in unstressed or stressed stage-specific RNA-seq replicates from Figure 1D. (B) Representative WT and Δ*BFD1*::*BFD1*^WT^-Ty vacuoles after 48 h of growth under unstressed or stressed conditions. Ty was immunostained with BB2 (magenta). Scale bar is 5 μm. (C) Immunoblot of lysates from unstressed or stressed Δ*BFD1*::*BFD1*^WT^-Ty parasites for the presence of BFD1-Ty or CDPK1. The expected molecular weight of BFD1 is 262.5 kDa. (D) Generation of a conditionally stabilized BFD1 overexpression strain. Addition of the stabilizing-ligand Shield-1 inhibits degradation of BFD1. (E) Representative images of Δ*BFD1*/*DD-BFD1*-*Ty* parasites grown in standard medium with vehicle (left) or 3 μM Shield-1 (right) added. After 4 days of growth, parasites grown in vehicle lyse the host cell monolayer, while parasites treated with Shield-1 continue to grow intracellularly. Scale bar is 20 μm. (F) Timeline to assay differentiation following induction of BFD1 expression. After 4 h, standard medium was replenished adding vehicle or to 3 μM Shield-1. After an additional 48 h, differentiation was quantified by DBL positivity. (G) Representative vacuoles of Δ*BFD1*/*DD-BFD1*-*Ty* parasites grown for 48 h in standard medium with vehicle or 3 μM Shield-1 and stained for Ty or DBL. Scale bar is 10 μm. (H) Quantification of (G). Mean plotted for n = 3–4 biological replicates, with DBL-positive vacuoles counted from at least 100 vacuoles per replicate. ^∗∗∗∗^p < 0.0001, Student’s one-tailed t test. (I) RNA sequencing and differential expression (DE) analysis identified 1,998 genes as significantly upregulated (green) and 2,110 genes as significantly downregulated (red) in Δ*BFD1*/*DD-BFD1*-*Ty* parasites grown for 48 h in 3 μM Shield-1 compared to vehicle alone (adjusted p < 0.001). Of these, 585 and 655 genes changed 2-fold or more (dotted lines). (J) Comparison of significantly regulated genes observed under alkaline stress or BFD1 expression (adjusted p < 0.001, 2-fold change or greater; points below this threshold were not plotted). Pearson correlation is indicated. (K) Overlap between differentially expressed genes plotted in (J). See also Figure S5 and Data S4.

**Figure S5:**
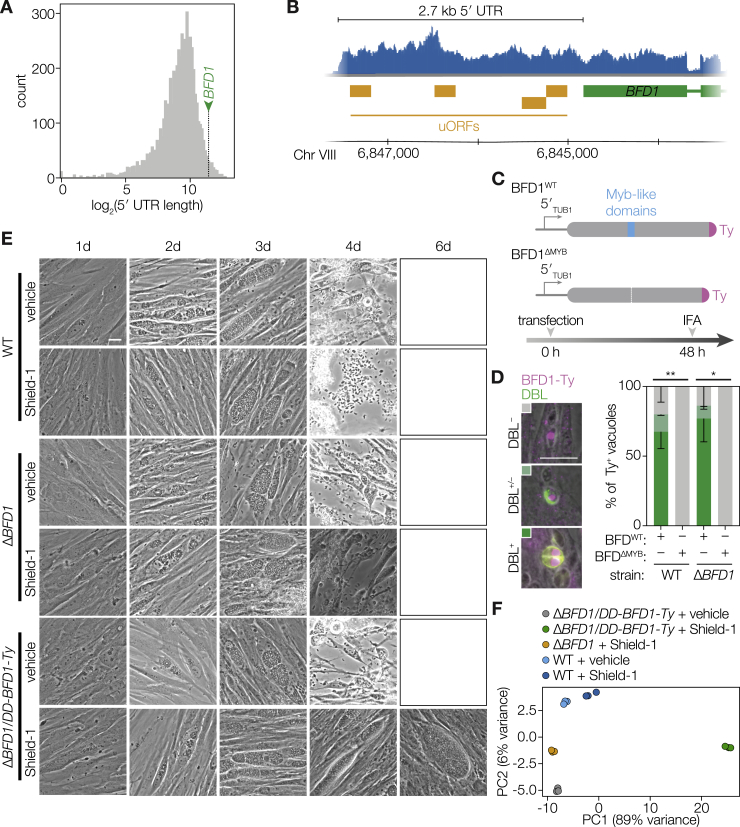
Conditional or Transient Overexpression of BFD1 Is Sufficient to Induce Differentiation in the Absence of Stress, Related to Figure 5 (A) Distribution of lengths of 5,699 previously annotated *Toxoplasma* 5′ UTRs. By manual annotation, the 5′ UTR of *BFD1* is 2,709 bp (green arrow), placing it in the 98^th^ percentile. (B) Diagram of the *BFD1* locus indicating RNA-seq read density from tachyzoites (blue) and indicating the position of upstream open reading frames (uORFs) in the 5′ UTR, and the *BFD1* coding sequence. (C**–**D) Constructs and experimental workflow for transient expression of BFD1. Epitope-tagged cDNA versions of wild-type BFD1 (*BFD1*^WT^) or BFD1 lacking its DNA-binding domains (BFD1^ΔMYB^) are under the regulation of the *TUB1* promoter (C). Parasites were immuno-labeled for Ty (magenta) and for differentiation with FITC-conjugated DBL (green) and differentiation in WT or Δ*BFD1* parasites 48 h after transient overexpression of BFD1^WT^ or BFD1^ΔMYB^ was quantified. Scale bar is 10 μm. Ty^+^ vacuoles were identified and then scored for DBL positivity as shown in representative images. Mean ± SD is plotted for n = 2 independent replicates, 17–61 vacuoles counted per replicate; ^∗^*p-value* < 0.05, ^∗∗^*p-value* < 0.01; Student’s one-tailed t test (D). (E) Representative images of WT, Δ*BFD1,* or Δ*BFD1*/*DD-BFD1*-*Ty* parasites grown in standard medium supplemented with vehicle or 3 μM Shield-1. After 4 days of growth, all host monolayers had been lysed except for the Shield-1-treated Δ*BFD1*/*DD-BFD1*-*Ty* cultures in which parasites continued replicating intracellularly. Scale bar is 10 μm. (F) Principal component analysis of RNA-seq of WT, Δ*BFD1,* or Δ*BFD1*/*DD-BFD1*-*Ty* parasites grown in media containing 3 μM Shield-1 or vehicle alone.

Our stage-specific transcriptional analysis found that *BFD1* mRNA levels are constant across different stages and present at a similar level to genes whose protein products are found in tachyzoites (Figure 5A). However, BFD1 protein, detected from the epitope-tagged allele in the complemented strain (Δ*BFD1*::*BFD1*^WT^*-Ty*), was only observed by immunofluorescence or immunoblot under stress conditions (Figures 5B and 5C). This observation suggests stress-dependent translation of BFD1. Inspection of the *BFD1* locus revealed an unusually long 5′ untranslated region (UTR), which at 2.7 kb in length falls in the 98^th^ percentile of all annotated 5′ UTRs in the *Toxoplasma* genome (Figure S5A). The 5′ UTR of *BFD1* encodes four untranslated open reading frames, 225–273 nucleotides long (Figure S5B) that, like UTR length, characterize many post-transcriptionally regulated genes (Hinnebusch et al., 2016).

The stage-specific presence of BFD1 raised the possibility this factor alone could be sufficient to drive differentiation if expressed in tachyzoites. Transient transfection with a construct constitutively expressing full-length BFD1 was sufficient to induce differentiation in over 60% of WT or Δ*BFD1* parasites, while expression of a mutant lacking the DNA-binding domains failed to induce differentiation in either genotype (Figures S5C and S5D). To investigate the effects of *BFD1* expression in greater detail, we complemented Δ*BFD1* with a regulatable version of *BFD1* (Δ*BFD1*/*DD-BFD1-Ty*, Figure 5D). In this strain, BFD1 is constitutively degraded after translation until Shield-1 is added to the culture media, stabilizing the degradation domain, and causing the accumulation of the transgene in the parasite (Banaszynski et al., 2006). Culturing Δ*BFD1*/*DD-BFD1-Ty* in media containing Shield-1 prevented lysis of the monolayers coinciding with the appearance of large cyst-like vacuoles (Figure 5E). Such changes were not observed with vehicle treatment, or Shield-1 treatment of Δ*BFD1* or WT parasites (Figure S5E). Shield-1 treatment led to robust nuclear DD-BFD1-Ty accumulation and was sufficient to induce differentiation in over 95% of parasites in the absence of any additional stress (Figures 5F–5H).

### Conditional Expression of BFD1 Recapitulates the Bradyzoite Transcriptional Program

To determine the extent of transcriptomic changes attributable to BFD1 activity, we sequenced the transcriptomes of WT, Δ*BFD1*, and Δ*BFD1/DD-BFD1-Ty* parasites in the presence or absence of Shield-1. Principal component analysis suggested Δ*BFD1/DD-BFD1-Ty* parasites treated with vehicle are similar to the parental Δ*BFD1* strain, while stabilization of BFD1 with Shield-1 was responsible for most of the variance between samples (Figure S5F). We identified 585 genes as upregulated and 655 genes as downregulated 2-fold or more in induced parasites, including many canonical stage-specific genes (Figure 5I; Data S4). 76% of upregulated genes and 45% of downregulated genes were previously identified as induced in WT parasites under alkaline stress (Figures 5J and 5K), including the canonical bradyzoite markers *BAG1*, *LDH2*, and *ENO1*.

### BFD1 Preferentially Binds at the Transcriptional Start Sites of Differentially Regulated Genes

**Figure 6:**
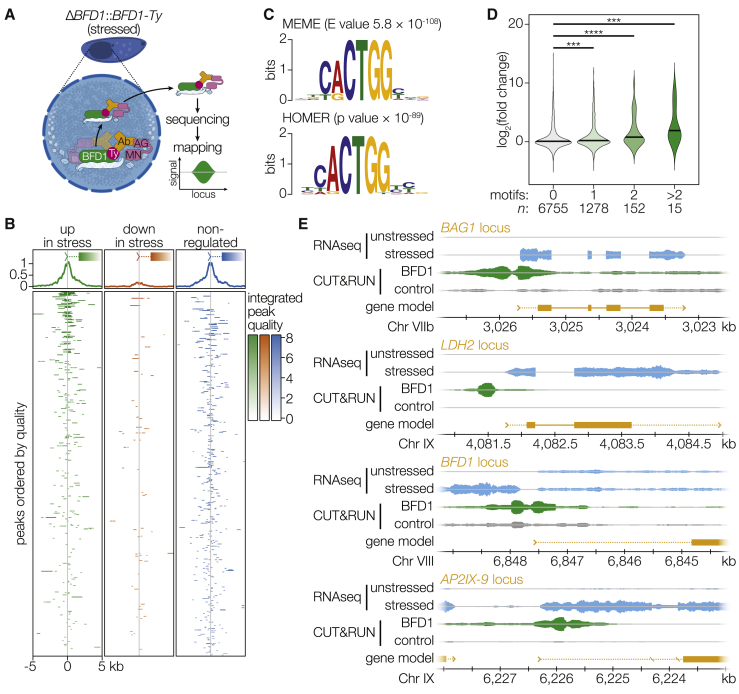
BFD1 Binds to Transcriptional Start Sites of Differentially Regulated Genes (A) Overview of the CUT&RUN protocol. Antibody-directed MNase activity preferentially creates short fragments inclusive of the region bound by the protein of interest. These short fragments diffuse out of the nucleus and can then be enriched and sequenced. (B) Distribution of BFD1 peaks ordered according to their quality score, plotted relative to the nearest transcriptional start site (TSS). Each row is a single gene with one or more associated BFD1 peaks. Genes are categorized according to their differential expression in stage-specific RNA sequencing. (C) MEME or HOMER motifs significantly enriched at BFD1 binding sites. (D) Genes with BFD1 binding motifs within a −443 to +494 bp window of their TSS tend to be upregulated following alkaline stress. Distribution of fold changes for genes in each category. ^∗∗∗^p < 0.001, ^∗∗∗∗^p < 0.0001, Mann-Whitney test. (E) Representative loci indicating binding of BFD1 upstream of many of the most differentially expressed genes and its own transcriptional start site. See also Data S5.

To identify the binding profile of BFD1 genome-wide, we adapted CUT&RUN to *Toxoplasma* (Skene and Henikoff, 2017) (STAR Methods). CUT&RUN targets the activity of micrococcal nuclease to sites of a target DNA-associated protein via antibody binding to generate short fragments of genomic DNA spanning the binding sites of a protein of interest (Figure 6A). These fragments diffuse out of isolated nuclei and can be enriched and sequenced. Applying this technique to alkaline-stressed Δ*BFD1*::*BFD1*^WT^*-Ty* parasites, we identified 815 BFD1 peaks throughout the genome, of which 652 could be unambiguously assigned to 509 genes. BFD1 binding sites are preferentially found near transcriptional start sites, and higher-quality peaks were more frequently associated with genes differentially regulated under alkaline stress (Figure 6B). Motif enrichment analysis using MEME (Bailey and Elkan, 1994) or HOMER (Heinz et al., 2010) each identified analogous motifs with high confidence (Figure 6C; Data S6). The presence of a binding motif is predictive of differential regulation under alkaline stress, and the number of upstream binding motifs correlates with the degree of differential expression seen (Figure 6D). BFD1 peaks were found upstream of many of the most differentially regulated genes in bradyzoites, including the canonical stage-specific genes *BAG1*, *LDH2*, and *ENO1* (Figure 6E). BFD1 also appears to bind upstream of its own promoter, in addition to the promoter of the known early bradyzoite transcription factor AP2IX-9.

## Discussion

Differentiation from tachyzoites to bradyzoites establishes chronic *Toxoplasma* infection. However, the molecular pathways regulating this transition have remained unclear, despite evidence that disparate inputs (heat shock, alkaline stress, and nutrient starvation) converge on a common transcriptional program. Using bulk and scRNA-seq, we characterize differentiation in unprecedented detail. Through Cas9-mediated genetic screens, we identify a single gene (*BFD1*) as indispensable for differentiation. Δ*BFD1* parasites grow normally under standard conditions but fail to differentiate under all induction conditions tested and are unable to form cysts in mice—defects reversed by complementation. BFD1 binds near the transcriptional start sites of many genes upregulated during differentiation, and its conditional expression is sufficient to induce differentiation in the absence of stress, recapitulating many transcriptional changes associated with stress-induced differentiation. Taken together, our results demonstrate that BFD1 operates as a master regulator of bradyzoite formation in *Toxoplasma*.

By profiling FACS-enriched differentiated populations, we captured transcriptional differences between tachyzoites and bradyzoites with sensitivity and dynamic range, uncovering differences previously masked by profiling mixed populations (Buchholz et al., 2011, Fritz et al., 2012, Pittman et al., 2014, Ramakrishnan et al., 2019). Differentially expressed genes reflect a combination of factors, including nutrient availability and general stress responses, in addition to the bradyzoite differentiation program. We note that 58% of genes identified as downregulated in our stage-specific RNA-seq are cell-cycle regulated, suggesting that changes in replication rate are responsible for many of the transcriptional differences observed at the population level (Data S1; Data S3).

A single family of DNA-binding proteins, the ApiAP2s, has been investigated for their role as *Toxoplasma* transcription factors and mediators of differentiation. While the phenotypes associated with many ApiAP2 mutants are striking, no single gene knockout has resulted in a complete block in differentiation, leading to the assumption that multiple transcription factors regulate *Toxoplasma* bradyzoite development (Jeffers et al., 2018). By screening a wider range of putative nucleic acid binding proteins, including those containing well-conserved DNA-binding motifs such as zinc finger and Myb-like domains, we discovered that inactivation of *BFD1* completely ablates bradyzoite formation. The screen results suggest a slightly increased fitness for *BFD1-*deficient parasites, consistent with the predicted advantage of not committing to differentiation at a rate of 1%–5% per lytic cycle. The role of BFD1 as a master regulator of differentiation does not preclude important functions for ApiAP2 proteins as downstream mediators of the bradyzoite program, and additional work is needed to characterize the precise hierarchy of transcriptional regulation. *Toxoplasma* encodes 13 other proteins containing SANT/Myb-like domains, suggesting the existence of a second extensive transcription factor family. Myb-domain-containing proteins are widespread among eukaryotes and have been implicated in the regulation of encystation in *Entamoeba* and *Giardia*, along with a wide variety of stress responses in plants (Ambawat et al., 2013, Ehrenkaufer et al., 2009, Sun et al., 2002). In humans, c-Myb is thought to function as a pioneer transcription factor, binding to chromatin and recruiting histone acetyltransferases to commit cells to specific hematopoietic lineages (Fuglerud et al., 2018, Sandberg et al., 2005). Among apicomplexan parasites, a Myb-domain-containing protein has been identified as important for erythrocytic growth of *Plasmodium falciparum*, suggesting that other family members likely play important roles throughout the phylum (Boschet et al., 2004, Gissot et al., 2005).

scRNA-seq enables profiling of thousands of cells across asynchronous processes and has been successfully used to examine commitment to sexual differentiation in *Plasmodium* spp. (Bancells et al., 2019, Poran et al., 2017). Implementing these approaches in *Toxoplasma* retained information about cell-cycle residency and the relative timing of gene expression that is lost in bulk analyses. Moreover, scRNA-seq allowed us to identify markers specific to actively replicating bradyzoites and genes expressed during the earliest stages of differentiation (Data S3). This detailed view of differentiation revealed that *BFD1* knockout parasites progress normally throughout the tachyzoite cycle but fail to initiate bradyzoite differentiation following alkaline stress.

Our transcriptional profiling reveals that *BFD1* is expressed in the 67^th^ percentile in tachyzoites, which is corroborated by other RNA-seq datasets (Melo et al., 2013, Ramakrishnan et al., 2019). *BFD1* transcription therefore does not appear to be stage specific despite a modest 1.5- to 3.6-fold upregulation in bradyzoites observed by bulk RNA-seq or scRNA-seq—the former below our cutoff for significance (Data S1; Data S3). However, expression of an epitope-tagged version of *BFD1* from the endogenous locus was only observed under stress conditions by immunofluorescence or by western blot, suggesting post-transcriptional regulation. In other eukaryotes, long 5′ UTRs containing small upstream open reading frames—as observed for *BFD1*—are the locus of translational regulation (Hinnebusch et al., 2016). Preferential translation of some transcripts under stress conditions has been reported in *Toxoplasma*, and mutations in RNA-binding proteins have resulted in severe differentiation defects, suggesting an important role for translational control during differentiation (Gissot et al., 2013, Holmes et al., 2014, Narasimhan et al., 2008).

Conditional expression of BFD1 is sufficient to induce differentiation in the absence of stress and recapitulates many of the transcriptional changes observed following alkaline-stress-induced differentiation including upregulation of the canonical stage-specific genes *BAG1*, *LDH2*, and *ENO1* and downregulation of *SAG1*. Other putative transcription factors, including several ApiAP2s, are also regulated by BFD1, implying a hierarchy of differentiation-promoting factors (Data S4). The core bradyzoite transcriptional program is difficult to define given the poor agreement between various studies of differentiation. Taking the intersection of all 2,485 genes identified as upregulated in bradyzoites in at least one of three recent studies and our own, only 99 genes are consistently identified (Behnke et al., 2008, Fritz et al., 2012, Ramakrishnan et al., 2019). Conditional expression of BFD1 is sufficient to significantly upregulate 90 of these 99 core genes, with 73 increasing expression by more than 2-fold (Data S1; Data S6). Many genes differentially upregulated under alkaline stress are not impacted by BFD1 expression, suggesting that these genes represent the transcriptional response to alkaline stress or altered nutritional conditions and are not truly stage-specific.

Adaptation of CUT&RUN to *Toxoplasma* enabled us to map the distribution of BFD1 binding sites across the genome. BFD1 binds preferentially at transcription start sites, particularly those of differentially regulated genes. The enrichment of BFD1 peaks upstream of differentially expressed genes suggests BFD1 directly drives some of the transcriptional differences between tachyzoites and bradyzoites. Future studies identifying BFD1 accessory factors and additional determinants of binding specificity will further define the characteristics of bradyzoite-specific promoters.

As a necessary and sufficient regulator of differentiation, *BFD1* provides a focal point for elucidating the molecular mechanisms underlying differentiation. In *Plasmodium*, identification of AP2-G as the master transcriptional regulator of gametogenesis has permitted placement of multiple genes observed to affect sexual differentiation into a unified regulatory framework, and allowed directed investigation of their mechanisms of action (Josling et al., 2018). The ability to induce synchronized sexual differentiation through conditional overexpression of AP2-G has allowed finer temporal mapping of the gene expression changes that accompany gametogenesis (Kent et al., 2018), and similar studies of *Toxoplasma* differentiation are now possible using our conditionally stabilized *BFD1* strain. The inability of Δ*BFD1* parasites to form brain cysts in mice suggests that Δ*BFD1* parasites may represent an ideal attenuated vaccine strain, capable of proliferating robustly yet unable to enter a chronic state. Collectively, modulation of BFD1 holds substantial clinical and biotechnological potential, as chronic infection represents a major barrier to both the treatment of *Toxoplasma* and its use in delivery of heterologous antigens and protein-based therapeutics.

## STAR★Methods

### Key Resources Table

**Table:** 

REAGENT or RESOURCE	SOURCE	IDENTIFIER
**Antibodies**		

Anti-Ty1 antibody (BB2), mouse	(Bastin et al., 1996)	N/A
Anti-GAP45, rabbit	(Plattner et al., 2008)	N/A
Anti-SAG2Y, rabbit	(Saeij et al., 2008)	N/A
Anti-SAG1, mouse	(Burg et al., 1988)	N/A
Anti-mouse 488	Life Technologies	Cat#A32723; RRID: AB_2633275
Anti-rabbit 594	Life Technologies	Cat#A11037; RRID: AB_2534095
Anti-mouse 594	Life Technologies	Cat#A11005; RRID: AB_2534073
Anti-mouse 647	Life Technologies	Cat#A32728; RRID: AB_2633277
Anti-CDPK1, guinea pig	Covance	Custom antibody
Anti-guinea pig IRDye 800	LICOR Biosciences	Cat#926-32411; RRID: AB_2814905
Anti-mouse IRDye 800	LICOR Biosciences	Cat#926-32210; RRID: AB_2687825
Mouse IgG Isotype Control	Thermo Fisher Scientific	Cat#14-4714-82; RRID: AB_470111
Fluorescein labeled Dolichos Biflorus Agglutinin (DBL)	Vector Laboratories	Cat#FL-1031; RRID: AB_2336394

**Bacterial and Virus Strains**		

E. cloni electrocompetent cells	Lucigen	Cat#60117-1
NEB 5-alpha competent cells	NEB	Cat#C2987I
JM101 competent cells	Agilent	Cat#200234

**Chemicals, Peptides, and Recombinant Proteins**		

Gentamicin	Thermo Fisher Scientific	Cat#15710072
Hoechst 33258 (CAS 23491-45-4)	Santa Cruz	Cat#sc-394049
HEPES	Sigma-Aldrich	Cat#H9897
Pyrimethamine	Sigma-Aldrich	Cat#46706-250MG
Compound 1	(Donald et al., 2002)	N/A
Mycophenolic acid	Sigma-Aldrich	Cat#M3536-50MG
Xanthine	Sigma-Aldrich	Cat#X4002-1G
6-thioxanthine	Sigma-Aldrich	Cat#T8125
TRIzol	Life Technologies	Cat#15596026
TRIzol LS	Life Technologies	Cat#10296010
Chloroform	VWR	Cat#AA22920-K2
Shield-1	Takara Bio	Cat#632189
Glycoblue	Life Technologies	Cat#AM9515
Glycogen	Life Technologies	Cat#R0561
Isopropyl-β-D-1-thiogalactopyranoside	Thermo Fisher Scientific	Cat#15529-019
chicken egg white lysozyme	Sigma-Aldrich	Cat#L3790
Imidazole	Sigma-Aldrich	Cat#I2399
PMSF Protease Inhibitor	Sigma-Aldrich	Cat#10837091001
Zwittergent 3-10 Detergent	Millipore Sigma	Cat#693021
IPTG	Thermo Fisher Scientific	Cat#15529-019
β-mercaptoethanol	Sigma-Aldrich	Cat#M6250
Magnesium sulfate heptahydrate	Sigma-Aldrich	Cat#230391
Spermidine	Sigma-Aldrich	Cat#S2626
IGEPAL CA-630	Sigma-Aldrich	Cat#I3021
PEG4000	EMD Millipore	Cat#25322-68-3
BSA	Sigma-Aldrich	Cat#A7030
RNase A	Thermo Fisher Scientific	Cat#EN0531
Prolong Diamond	Life Technologies	Cat#P36961
DNaseI	NEB	Cat#M0303L
TURBO DNA-free kit	Thermo Fisher Scientific	Cat#AM1907
BsaI	NEB	Cat#R0535L
AseI	NEB	Cat#R0526L
Exonuclease I	NEB	Cat#M0293M
Proteinase K	Life Technologies	Cat#25530-049
Phenol:Chloroform:Isoamyl solution	Invitrogen	Cat#15593031
T4 polynucleotide kinase	NEB	Cat#B0202S
T4 DNA polymerase	NEB	Cat#18005-017
Taq DNA polymerase	Thermo Fisher Scientific	Cat#EP0401
Quick ligase	Enzymatics	Cat#L6030-HC-L
iProof High-Fidelity DNA polymerase	Bio-Rad	Cat#1725301
cOmplete, Mini, EDTA-free Protease Inhibitor Cocktail tablet	Roche	Cat#11836170001

**Critical Commercial Assays**		

QuBit dsDNA HS Assay	Life Technologies	Cat#Q32851, Cat#Q32856
DNeasy Blood and Tissue kit	QIAGEN	Cat#69506
ZR Plasmid Miniprep	Zymo Research	Cat#D4016
ZymoPURE Plasmid Maxiprep Kit	Zymo Research	Cat#D4203
Zymoclean Gel DNA Recovery Kit	Zymo Research	Cat#D4002
Nextera XT DNA Library Prep Kit	Illumina	Cat#FC-131-1024
SMARTseq v2 Low Input RNA Kit	Takara Bio	Cat#634889
MiSeq Reagent Kit V2 (50 cycles)	Illumina	Cat#MS-102-2001
NextSeq 500 V2 High Output Kit (75 cycles)	Illumina	Cat#FC-404-2005
NEBNext Ultra II Directional RNA Library Prep Kit	NEB	Cat#E7760L
TruSeq DNA Single Indexes Set A	Illumina	Cat#20015960
Q5 2x MasterMix	NEB	Cat#M0492L
KAPA HiFi PCR Mix	Kapa Biosystems	Cat#KK2602
Gibson 2x MasterMix	NEB	Cat#E2611L

**Deposited Data**		

Next-generation sequencing data generated from genetic screens, RNA-sequencing, and CUT&RUN experiments	Gene Expression Omnibus	GEO: GSE132250

**Experimental Models: Cell Lines**		

Human foreskin fibroblasts (HFFs)	ATCC	SCRC-1041

**Experimental Models: Organisms/Strains**		

*Toxoplasma gondii* ME49 pGRA1-dsRed2.0 pBAG1-mNeonGreen pTUB1-Cas9 (bradyzoite reporter strain)	This paper	N/A
*T. gondii* ME49 pGRA1-dsRed2.0 pBAG1-mNeonGreen pTUB1-Cas9 BFD1-frameshift	This paper	N/A
*T. gondii* ME49Δ*KU80* (WT)	This paper	N/A
*T. gondii* ME49Δ*KU80*Δ*BFD1* (Δ*BFD1*)	This paper	N/A
*T. gondii* ME49Δ*KU80*Δ*BFD1*::*BFD1*^WT^-*Ty* (Δ*BFD1*::*BFD1*^WT^-*Ty*)	This paper	N/A
*T. gondii* ME49Δ*KU80*ΔBFD1::BFD1^ΔMYB^-Ty (ΔBFD1::BFD1^ΔMYB^-Ty)	This paper	N/A
*T. gondii* ME49Δ*KU80*Δ*BFD1*/*HXGPRT*::p*TUB1-DD-BFD1-Ty* (Δ*BFD1*/*DD-BFD1-Ty*)	This paper	N/A
*Mus musculus* CD-1	Charles River	Strain code: 022
*M. musculus* CBA/J	Jackson Labs	Strain Code: 000656

**Oligonucleotides**		

Refer to Data S6		

**Recombinant DNA**		

Refer to Data S6		

**Software and Algorithms**		

STAR v. 2.6.1	(Dobin et al., 2013)	https://github.com/alexdobin/STAR
Bowtie2 v. 2.3.4.1	(Langmead and Salzberg, 2012)	http://bowtie-bio.sourceforge.net/bowtie2/index.shtml
Prism 8	GraphPad	https://graphpad.com
The R Project for Statistical Computing v. 3.5.0		www.r-project.org
RStudio	RStudio	Rstudio.com
DESeq2 v. 1.26	(Love et al., 2014)	https://github.com/mikelove/DESeq2
Seurat v. 2.3.4	(Butler et al., 2018)	https://github.com/satijalab/seurat
Tidyverse		https://tidyverse.tidyverse.org/
FIJI	(Schindelin et al., 2012)	https://github.com/fiji
DropSeqTools v1.0		https://github.com/broadinstitute/Drop-seq/
IGV	(Robinson et al., 2011)	http://software.broadinstitute.org/software/igv/download
FCS Express 6	De Novo Software	Denovosoftware.com
SnapGene	SnapGene	https://www.snapgene.com/
ToxoDB	(Gajria et al., 2008)	ToxoDB.org
Trim_galore v 0.4.1	Babraham Bioinformatics	https://www.bioinformatics.babraham.ac.uk/projects/trim_galore/
FigTree v. 1.4.4		https://github.com/rambaut/figtree/releases
MACS2 v. 2.1.1.20160309	(Feng et al., 2012)	https://github.com/taoliu/MACS
MEME v. 4.11.4	(Bailey and Elkan, 1994)	http://meme-suite.org
Homer v. 4.10	(Heinz et al., 2010)	http://homer.ucsd.edu/homer/
BioVenn	(Hulsen et al., 2008)	http://biovenn.nl
**Other**		

Filter holders	VWR	Cat#28163-089
Whatman Nuclepore Hydrophilic Membrane filter, 3 μm	VWR	Cat#28158-624
Whatman Nuclepore Hydrophilic Membrane filter, 5 μm	VWR	Cat#28158-704
5 μm syringe filters	Thermo Fisher Scientific	Cat#SLSV025LS
Phase lock heavy	Quantabio	Cat#2302830
Phase lock light	Quantabio	Cat#2302820
Ampure RNAClean XP bead solution	Beckman Coulter	Cat#A63987
Barcoded mRNA capture beads	Chemgenes	Cat#MACOSKO-2011-10
Ni-NTA Agarose	QIAGEN	Cat#30230
Slide-A-Lyzer Dialysis Cassettes	Thermo Fisher Scientific	Cat#66003
BioMag Plus Concanavalin A coated magnetic beads	Polysciences	Cat#86057

### Lead Contact and Materials Availability

Further information and requests for resources and reagents should be directed to and will be fulfilled by the Lead Contact, Sebastian Lourido (lourido@wi.mit.edu). All unique/stable reagents generated in this study are available from the Lead Contact with a completed Materials Transfer Agreement.

### Experimental Model and Subject Details

#### Parasite and host cell culture

*T. gondii* parasites were grown in human foreskin fibroblasts (HFFs) maintained in DMEM (GIBCO) supplemented with 3% inactivated fetal serum (IFS) and 10 μg/mL gentamicin (Thermo Fisher Scientific), referred to as standard medium. If HFFs were to be used in bradyzoite experiments, host cells were maintained exclusively in DMEM supplemented with 10% IFS and 10 μg/mL gentamicin prior to infection. Alkaline stress medium consists of RPMI 1640 (Sigma), supplemented with 1% IFS and 10 μg/mL gentamicin, and buffered with 50 mM HEPES adjusted to pH 8.1 with 10 N NaOH. Compound 1 was used at 3 μM in standard medium (Radke et al., 2006). Shield-1 was used at 3 μM in standard medium (Banaszynski et al., 2006).

#### Mice

6-week-old female CD-1 (Charles River) or CBA/J (Jackson Labs) mice were purchased and maintained at the Whitehead Institute in facilities accredited by the Association for Assessment and Accreditation of Laboratory Animal Care. All protocols were approved by the Institutional Animal Care and Use Committee at the Massachusetts Institute of Technology. Mice were 7–8 weeks old at the beginning of the experiments.

### Method Details

#### Plasmids and primers

Oligos were ordered from IDT. All cloning was performed with Q5 2 × master mix (NEB) unless otherwise noted. Primers and plasmids used or generated in this study can be found in Data S6.

#### Parasite transfection

Parasites were mechanically released from host cells by scraping followed by passage through a 27-gauge needle. Parasites were pelleted at 400 × *g* for 10 min and resuspended in Cytomix (10  mM KPO_4_, 120  mM KCl, 150  mM CaCl_2_, 5  mM MgCl_2_, 25  mM HEPES, 2  mM EDTA), supplemented with 2  mM ATP and 5  mM glutathione, and combined with constructs being transfected to a final volume of 400 μL. Electroporation used an ECM 830 Square Wave electroporator (BTX) in 4 mm cuvettes with the following settings: 1.7 kV, 2 pulses, 176 μs pulse length, and 100 ms interval.

#### Parasite strain generation

##### Bradyzoite reporter strain

Starting with a robustly cyst-forming ME49 strain that constitutively expresses RFP (dsRed2.0) under the control of the *GRA1* promoter (Oldenhove et al., 2009), we inactivated the endogenous selectable marker *HXGPRT* through transfection with three gRNAs targeting the third, fourth and fifth exons. These gRNA expression vectors were assembled by annealing oligos P1/P2, P3/P4, and P5/P6, ligating into BsaI (NEB) digested pU6-Universal (AddGene #52694), and sequence verifying with P19 (Sidik et al., 2014). Transfected parasites were selected with standard media containing 300 μg/mL 6-thioxanthine and prepared with dialyzed IFS, and screened for large deletions with P7/P8 (Donald et al., 1996). This strain was made constitutively Cas9^+^ by co-transfection with pCas9-CAT (AddGene #80323) and pU6-Decoy (AddGene #80324) as described previously (Sidik et al., 2016). The strain was further transfected with ScaI (NEB) linearized pBAG1-mNeonGreen, which contains the promoter of BAG1 (1.22 kb upstream of the coding sequence ATG, amplified with primers P9/10) driving expression of mNeonGreen, and also contains a HXGPRT resistance cassette. We selected for integration with 25 μg/mL mycophenolic acid and 50 μg/mL xanthine (Behnke et al., 2008, Bohne and Roos, 1997). Note this plasmid contains two identical DHFR 3′ UTRs, and care had to be taken to avoid the loss of HXGPRT by recombination during growth in bacteria.

##### BFD1^frameshift^

A gRNA was designed targeting the first exon of *BFD1*. Oligos P89/P90 were annealed, Gibson-assembled into pU6-Universal, and sequenced verified with P19, generating plasmid pU6-BFD1-DHFR. Bradyzoite reporter strain parasites were transfected with 50 μg of AseI (NEB) linearized pU6-BFD1-DHFR, and selected with 3 μM pyrimethamine in standard medium the next day. After stabilization of the population, parasites were subcloned into 96-well plates at 3 parasites per well. Clonal strains isolated from single plaques were screened and sequenced for polymorphisms at the targeted site.

##### ME49ΔKU80

Two gRNAs were designed targeting regions immediately upstream or downstream of the *KU80* coding sequence. Oligos P11/12, P13/14, P15/16, P17/18 were annealed, Gibson-assembled into pU6-Universal, and sequence verified with P19. An early passage ME49 strain was transfected with 25 μg of each plasmid, and immediately subcloned into 96-well plates at 20 or 40 parasites per well to account for loss of viability during transfection. Clonal strains isolated from single plaques were screened for deletion of *KU80* with P20/21, which amplifies a band of ∼5.9 kb in wild-type parasites or ∼500 bp if *KU80* is excised. A single mixed population was identified from 225 clones tested, and further subcloned to isolate ME49Δ*KU80*. Loss of *KU80* was confirmed by complete sequencing of the locus and failure to amplify an internal fragment using P22/P23.

##### ME49ΔKU80ΔBFD1 (ΔBFD1)

Two gRNAs were designed targeting regions immediately upstream or downstream of *BFD1*. Oligos P24/25 and P26/27 were annealed, Gibson-assembled into pU6-Universal, and sequenced verified with P19. A repair template consisting of the *SAG1* promoter driving expression of mNeonGreen was amplified from p*SAG1*-mNeonGreen using primers P28/29 with 40 bp of homology to regions flanking the targeted sites. ME49Δ*KU80* was transfected with 50 μg of each gRNA and 10 μg of repair template. 5 days post-transfection parasites were sorted by green fluorescence and subcloned. Clonal strains isolated from single plaques were further characterized by sequencing the locus using P30/31 to confirm complete deletion of *BFD1*.

##### ME49ΔKU80ΔBFD1::BFD1^WT^-Ty and ME49ΔKU80ΔBFD1^ΔMYB^-Ty (ΔBFD1::BFD1^WT^-Ty and ΔBFD1^ΔMYB^-Ty)

Two gRNAs targeting either junction of the pSAG1-mNeonGreen cassette previously integrated at the *BFD1* locus to create ME49Δ*KU80*Δ*BFD1*. Oligos P91/P92 and P93/P94 were annealed, Gibson assembled into pU6-Universal, and sequence verified with P19. Repair plasmids encoding either full-length (pBFD1-BFD1^WT^-Ty) or Myb-domain deleted (pBFD1-BFD1^ΔMYB^-Ty) *BFD1* and homology to the native locus were constructed as follows. The sequence of *BFD1* was amplified from ME49 cDNA using primers P32/P33. *BFD1* fragments were combined by Gibson Assembly (NEB) with 1 kb of sequence immediately upstream of the *BFD1* coding sequence (amplified with P95/P96) and the native *BFD1* 3′ UTR (∼1.1 kb amplified with P38/39), and verified by Sanger sequencing with oligos P79–P85. To amplify *BFD1* lacking the DNA-binding domain (removing amino acids 921–1019), primers P32/P34 and P35/P33 were used and this fragments was used in place of the P32/P33 fragment. In both cases, the reverse primers for the *BFD1* cDNA append a C-terminal Ty epitope tag in-frame. ME49Δ*KU80*Δ*BFD1* parasites were transfected with 50 μg of each gRNA plasmid, along with 50 μg of NotI/PacI (NEB) digested repair plasmid pBFD1-BFD1_wt-Ty or pBFD1-BFD1_ΔBFD1-Ty, and subcloned in 96 well plates after one passage. Clonal strains isolated from single plaques were screened for correct integration using P98/P99 and P85/P31 to check the 5′ and 3′ prime junctions, respectively.

##### ME49ΔKU80ΔBFD1/HXGPRT::pTUB1-DD-BFD1-Ty (ΔBFD1/DD-BFD1-Ty)

A gRNA targeting the third exon of *HXGPRT* was assembled as described for the bradyzoite reporter strain, using P1/P2. An N-terminally DD-tagged cDNA copy of *BFD1* driven by the TUB1 promoter (pTUB1-DD-BFD1-Ty1) was assembled as follows. The promoter of *TUB1* and upstream homology to the *HXGPRT* integration site were amplified using P37/P100, the DD domain was amplified by P101/P102, the *BFD1* cDNA C-terminally epitope-tagged with Ty was amplified using P103/P33, and the native *BFD1* 3′ UTR and downstream homology to the *HXGPRT* integration site were amplified using P38/P39. These fragments were Gibson assembled together and verified by Sanger sequencing with oligos P79–P85 and P97. ME49Δ*KU80*Δ*BFD1* parasites were transfected with 50 μg *HXGPRT*-targeting gRNA and 50 μg PacI/NotI (NEB) linearized pTUB1-DD-BFD1-Ty. After one passage, parasites were selected with standard media prepared with dialyzed IFS and supplemented with 300 μg/mL 6-thioxanthine. After two weeks of selection, parasites were subcloned in 96-well plates, and screened for correct integration using P7/P104 and P85/P8 to check the 5′ and 3′ junctions, respectively.

#### Immunoblotting

Δ*BFD1*::*BFD1*^WT^-Ty parasites were grown in standard media for 24 h before being shifted to alkaline stress conditions (alkaline stress media, ambient CO_2_) or kept under unstressed conditions. Unstressed parasites were scraped, serially passaged through 27 and 30-gauge needles, and filtered through a 5 μm filter after an additional 24 h of growth (48 h post infection). Alkaline stressed parasites were harvested similarly, but after 48 h of growth under alkaline stress (72 h post infection). Parasite pellets were resuspended in 1 × Laemmli buffer (diluted from 5 × buffer containing 10% SDS, 50% glycerol, 300 mM Tris HCl pH 6.8, 0.05% bromophenol blue) with 1% final volume β-mercaptoethanol and boiled for 10 min. Samples were run on a 4%–15% SDS-PAGE gel (BioRad), and transferred overnight onto a nitrocellulose membrane in transfer buffer containing 0.1% SDS (25 mM TrisHCl, 192 mM glycine, 0.1% SDS, 20% methanol). Blocking and all subsequent antibody incubations were performed in 5% milk in 0.5 × TBS-T (1L PBS, 250 μL Tween-20). Primary and secondary antibody incubations were done for 1 h rocking at room temperature, with three TBS-T washes between primary and secondary and between secondary and imaging. Imaging was performed using a LI-COR Odyssey CLx.

#### Plaque assays

Parasites were inoculated into 6 well plates of HFFs maintained in DMEM supplemented with 10% IFS and 10 μg/mL gentamicin, and allowed to grow undisturbed for 14 days. Plates were washed with PBS, and fixed for 10 min at room temperature with 100% ethanol. Staining was performed for 5 min at room temperature with 2 mL crystal violet solution per well (12.5 g crystal violet, 125 mL 100% ethanol, 500 mL 1% ammonium oxalate), followed by two washes with water and drying overnight.

#### Immunofluorescence assays

HFFs were grown on coverslips for 2–3 days before inoculation with *Toxoplasma*. Coverslips were fixed with 4% formaldehyde for 20 min, permeabilized with 1% Triton X-100 for 8 min, and blocked (5% normal goat serum and 5% IFS in PBS for at least 15 min (Figures 1, 2, 5, and S5). Alternatively, fixation was done using ice-cold 100% methanol for 2 min, without further permeabilization, followed by blocking as above (Figure S4). All primary and secondary antibody incubations were performed for 1 h, with coverslips inverted on 50 μL of antibody dilutions in blocking buffer on Parafilm in a humidified chamber. Three washes with PBS were performed after each step. Coverslips were mounted on 5 μL of Prolong Diamond (Thermo Fisher) and set for 30 min at 37°C or overnight at room temperature. DBL-488 (Vector Labs) was used at 1:500. Mouse-anti-Ty antibody (BB2) was used at 1:1,000 (Bastin et al., 1996). Rabbit-anti-GAP45 was a gift from Dominique Soldati (University of Geneva) and was used at 1:1,000 (Plattner et al., 2008). Rabbit-anti-SAG2Y was used at 1:2,000 (Saeij et al., 2008). Mouse-anti-SAG1 (DG52) was used at 1:500 (Burg et al., 1988). Hoechst 33258 (Santa Cruz) was used at 1:2,000. Secondary antibodies labeled with Alexa Fluor 488, 594 or 647 (Thermo Fisher) were used at 1:1,000.

#### Quantification of gene disruption

Bradyzoite reporter strain parasites were transfected with 50 μg of AseI (NEB) linearized pU6-SAG1-DHFR (Addgene #80322), encoding a gRNA targeting *SAG1*. Selection with 3 μM pyrimethamine in standard medium was initiated the next day, and drug-resistant pools were inoculated onto coverslips two passages (five days) after transfection. Coverslips were fixed 24 h later with methanol, and stained for SAG1 to quantify disruption rates, relative to an untransfected control. GAP45 was used as a counterstain. Gene disruption rates were quantified before each screen to ensure Cas9 activity.

#### Endogenous tagging

To endogenously tag TGME49_312330 and TGME49_208740, ME49Δ*KU80* was co-transfected with 50 μg of pCas9-CAT and 50 μg of BsaI-linearized p312330-Ty or p208740-Ty. Selection with 3 μM pyrimethamine in standard medium was initiated the following day. Parasites were subcloned in 96-well plates, and isolated clones screened for successful integration using primers P86/88 or P87/88, respectively, and validated by Sanger sequencing.

#### Overexpression vectors of BFD1^WT^ and BFD1^ΔMYB^

*BFD1* was amplified from ME49 cDNA using primers P32/33, additionally adding a C-terminal Ty epitope tag To amplify *BFD1* lacking the DNA-binding domain (removing amino acids 921–1019), primers P32/34 and P35/P33 were used, similarly adding a Ty epitope. *BFD1* fragments were combined by Gibson Assembly (NEB) with the *TUB1* promoter (amplified with P36/37) and the native *BFD1* 3′ UTR (∼1.1 kb amplified with P38/39), and verified by Sanger sequencing with oligos P79–P85.

#### Phylogenetic analysis of BFD1

Protein sequences containing SANT/Myb-like domains were obtained for representative apicomplexan genomes from EupathDB based on their annotation with SMART domain SM00717. Domains from human c-Myb and CDC5L were used for comparison. Individual domains were extracted from each sequence and aligned using ClustalW, and the phylogenetic tree was generated by neighbor-joining (Figure S2B) (Larkin et al., 2007). Alignments were also prepared for the concatenated domain sequences for a subset of proteins, and Bootstrap values were calculated for 10,000 trials (Figure 2A). Visualizations were generated using FigTree (v1.4.4).

#### Assessment of brain cyst formation

##### CD-1 infections

Mice were randomly assigned to inoculation with either mock (PBS), or 500 tachyzoites of WT (ME49Δ*KU80*), Δ*BFD1*, or Δ*BFD1::BFD1*^WT^*-Ty* (n = 5 for mock injections and n = 15 for each infection). Brains were collected from moribund animals starting 2 weeks post infection. All surviving mice were sacrificed at 5 weeks post infection.

##### CBA/J infections

Mice were randomly assigned to either low (100 tachyzoites) or high (2,000 tachyzoites) inoculation i.p. with WT (ME49Δ*KU80*), Δ*BFD1*, or Δ*BFD1::BFD1*^WT^*-Ty* (n = 5 mice per condition). Mice were sacrificed and brains collected at 2 weeks post inoculation.

##### *Ex vivo* cyst quantification

Brains were dissected into 2 mL PBS, and homogenized through repeated extrusion through an 18-gauge needle. 25 mL of PBS was added, and homogenates were spun for 5 min at 1,000 × *g*. The supernatant was discarded, and the homogenate was resuspended in 700 μL PBS, The final volume of homogenate (1–1.5 mL) was recorded. 100 μL of homogenate was added to 900 μL of ice-cold methanol, and fixed at room temperature for 5 min. Samples were then centrifuged for 5 min at 5,200 × *g* (conditions used for all subsequent centrifugations), supernatant decanted, washed with 1 mL PBS, and centrifuged again. Following aspiration of supernatant, brains were resuspended in 500 μL of primary staining solution (1:150 DBL-488, 1:1,000 guinea pig anti-CDPK1 in PBS) and placed at 4°C, rotating overnight. The following day, homogenates were centrifuged, supernatant decanted, and washed with 1 mL PBS before resuspending in 500 μL of secondary staining solution (1:1,000 anti-guinea pig–594) and incubating at 25°C rotating for 1 h. Homogenates were again centrifuged, washed twice with 1 mL PBS, and resuspended in 1 mL PBS. For each sample, four 50 μL samples were randomly assigned positions in a clear-bottomed 96 well plate and quantified blindly at 10 × magnification using DBL positivity as a first criterion and CDPK1 positivity to confirm brain cyst identity. Cyst burdens were calculated from the mean of the four samples, multiplied by the appropriate dilution factor.

#### Guide RNA library assembly

The gRNA oligonucleotide library was synthesized by Agilent and resuspended at 1 ng/μL in water. All library amplifications were done using iProof (Bio-Rad), using 2.5 ng of the oligonucleotide pool as template per 50 μL reaction. Sublibraries were amplified using primers P40/41 for library 1 and P42/43 for library 2, and subsequently amplified with primers P44/45 for cloning. Amplified libraries were Gibson assembled into gel-extracted (Zymo) BsaI-digested pU6_Library_DHFR, dialyzed against water, and electroporated into E. cloni electrocompetent cells (Lucigen) according to manufacturer’s protocol. Coverage was assessed by dilution plating in comparison to a no-insert negative control. Libraries were maxiprepped (Zymo), and retransformed into chemically competent NEB 5-alpha (NEB) to improve yields for transfection. Both E. cloni and NEB 5-alpha libraries were sequenced to ensure diversity. Libraries were linearized with AseI, dialyzed 1 h against water, and divided into 50 μg aliquots. Guide RNAs against mNeonGreen were assembled separately by annealing primer pairs P46/47, P48/49, P50/51, P52/53, or P54/55 and Gibson assembling into gel-extracted, BsaI-digested pU6_Library_DHFR. Constructs were verified by sequencing with P19, and spiked into library aliquots at equimolar concentrations.

#### Cas9-mediated genetic screening

Reporter strain parasites were grown up in 10 × 15-cm plates per screen. 10 transfections were performed for each library as described above, with each transfection using 50 μg of library and 2.6 × 10^7^ parasites in 400 μL cytomix (Sidik et al., 2014). Transfections were pooled and split among 4 × 15-cm dishes. Medium was changed the following day to standard media supplemented with 3 μM pyrimethamine and 10 μg/mL DNaseI (Sigma-Aldrich). At each passage of the screen, plates were scraped and parasites were mechanically released with a 27-gauge needle. For the second passage of the screen, all parasites were passed into 4 × 15-cm plates without counting. All subsequent passages were performed at a multiplicity of infection (MOI) of 1 (6 × 10^6^ parasites per plate). Plates lysed every 2–3 days under unstressed growth in standard media supplemented with 3 μM pyrimethamine. At the fourth passage (14 days post-transfection), parasites were inoculated into seven 15-cm plates. After 4 h the medium was exchanged for 3 plates to standard medium supplemented with 3 μM pyrimethamine, and for the remaining 4 plates to alkaline stress medium. Unstressed parasites were passaged at an MOI of 1 every 2–3 days into one or two 15-cm plates, in standard medium supplemented with 3 μM pyrimethamine. Parasites under stressed conditions did not lyse the monolayers and were not passaged for the duration of the experiment, but the medium was exchanged every 2 days to fresh alkaline-stress medium. At each passage of unstressed parasites, 1–4 × 10^7^ parasites were frozen down. At 10 days post the initial media exchange, stressed populations were scraped, parasites were mechanically released, passed through a 3 μm filter, and sorted based on green fluorescence. Sorting was performed using a BD FACS Aria II. At final time points for stressed parasites, both bulk populations (2 × 10^5^ parasites) and mNG^+^-sorted populations (7 × 10^5^) were frozen. DNA was isolated using the QIAGEN Blood and Tissue kit, following the manufacturer’s protocol for blood cells. Integrated gRNAs were amplified using primers P56 and one of the barcoded primers P57–P76 in 50 μL reactions. Each reaction contained 200 ng or a maximum of 20 μL of template DNA. Amplicons were gel-extracted (Zymo), eluted in water, and quantified using the QuBit dsDNA HS kit (Thermo Fisher). Amplicons were pooled equally at a final concentration of 8 pM each and sequenced using a MiSeq v2 kit. Reads were 40 bp single-end and an 8 bp index. Custom sequencing primer (P77) and custom indexing primer P78 were used. Only perfect matches were considered when counting the number of times each gRNA occurred in sample and gRNAs not detected were assigned a pseudocount of 90% of the lowest detected gRNA in that sample. The phenotype or differentiation score for a gene was calculated by determining the mean log_2_-transformed fold change for all five gRNAs targeting that gene in the final sample compared to the input library. All analyses were done in R.

#### Stage-specific RNA-seq and analysis

Parasites were allowed to invade and replicate inside host cells for 24 h under standard conditions, and then switched to standard or alkaline-stress medium. For FACS, parasites were mechanically released from host cells using a 27- followed by a 30-gauge syringe needle, and passed through a 3 μm filter. At 24 and 48 h post medium change, ∼1 × 10^5^ unstressed mNG^–^ or stressed mNG^+^ parasites were sorted directly into TRIzol LS (Ambion) and frozen on dry ice. Sorting was done using a BD FACS Aria II, and visualization of events and gates using FCS Express 6. RNA was extracted by TRIzol-chloroform according to manufacturer’s protocol, DNaseI digested, and TRIzol-chloroform extracted again. RNA quality was assessed using a BioAnalyzer or Fragment analyzer. When possible, two samples were prepared per replicate and time point and treated as technical replicates in downstream processing. Libraries were generated using the SMARTseq low-input v4 kit, and sequenced on two lanes of a HiSeq 2000. Reads were 75 bp, paired-end. Alignment to the ToxoDB v. 36 assembly of the ME49 genome was done using STAR (v. 2.6.1) (Dobin et al., 2013). Differential expression analysis was done using the DESeq2 R package (v. 1.21.16) (Love et al., 2014). Additional entries into the ToxoDB v. 36 GFF and genome file were added to include the Cas9, dsRed2.0, and mNeonGreen reporter constructs, and are listed as appearing on scaffold “BW123456.” The cutoff for differential expression was an adjusted *p-value* < 0.001.

#### Single-cell RNA-seq and analysis

Single-cell suspensions of *Toxoplasma* were prepared by syringe release of parasites from host cells with a 27- followed by a 30-gauge needle, followed by filtering through a 5 μm filter and counting on a haemocytometer. Once the parasites were isolated and a single-cell suspension generated, we used Seq-Well, a massively-parallel, low-input scRNA-seq platform, to capture the transcriptomes of single parasites on barcoded mRNA capture beads. Full methods on implementation of this platform are available (Hughes et al., 2019) and are hosted on the Shalek Lab website (http://www.shaleklab.com). Briefly, 10,000–20,000 parasites were loaded onto a functionalized, polydimethylsiloxane (PDMS) array preloaded with uniquely-barcoded mRNA capture beads (Chemgenes). After parasites had settled into wells, the array was sealed with a functionalized polycarbonate membrane with 10 nm pore sizes, facilitating buffer exchange while confining biological macromolecules within each well. After membranes were hybridized to the arrays using heat, subsequent buffer exchange allowed parasite lysis and mRNA molecule hybridization to beads, and then bead were removed to proceed with reverse transcription. Following reverse transcription, the bead bound cDNA-mRNA hybrid complex underwent Exonuclease I treatment (NEB) to remove single-strand primers before proceeding with second strand synthesis (S3).

Following Exonuclease I treatment, beads were washed once with TE-SDS (0.5% SDS) solution and twice with TE-TW (0.01% Tween) solution. After the second TE-TW wash, the beads were solvated with 0.1 M NaOH for 5 min with end-over-end rotation to denature the mRNA-cDNA hybrid product on the bead. Following denaturing, the NaOH was immediately removed and the beads were washed once with 1 M TE, and then solvated with the (S3) mastermix consisting of 5 × maxima RT buffer, 30% PEG-8000 solution, 10 mM dNTPs, 1 mM dN-SMART oligo, Klenow Exo^–^, and deionized ultrapure water. S3 was performed for 1 h where the beads were incubated at 37°C with end-over-end rotation. Following the incubation, beads were subsequently washed twice with TE-TW solution, once with 1 M TE and once more with deionized ultrapure water before proceeding with whole-transcriptome amplification (WTA).

WTA was performed using KAPA HiFi PCR Mix (Kapa Biosystems). Specifically, a PCR Mastermix consisting of KAPA 5 × Mastermix, 100 μM ISPCR oligo, and nuclease-free water combined with 2,000 beads per reaction. For each sample/array, the total number of PCR reactions performed varied depending on the number of beads recovered following S3. PCR amplification was performed using the following cycling conditions: initial denaturation at 95°C for 3 min; 4 cycles of 98°C for 20 s, 65°C for 45 s, and 72°C for 3 min; followed by 12 cycles of 98°C for 20 s, 67°C for 20 s, and 72°C for 3 min; and a final extension of 72°C for 5 min. Following WTA, the PCR products were purified using Ampure SPRI beads (Beckman Coulter) at 0.6 × and then 0.8 × volumetric ratios. The quality and concentration of the purified product was determined using an Agilent 4200 TapeStation instrument.

After confirming the purified product met the necessary quality control thresholds, Next-Generation Sequencing (NGS) libraries were prepared. Using a Nextera XT sample preparation kit (Illumina), a total of 1 ng of WTA product was combined with buffer TD, buffer ATM, and incubated at 55°C for 5 min to tagment the WTA product. Critically, since the parasite WTA production had a smaller distribution compared to human clinical samples, which is what Seq-Well was originally designed for, we adjusted the volumetric ratio of cDNA Input:buffer TD:buffer ATM (CI:TD:ATM) to account for this (i.e., normal CI:TD:ATM ratio is 1:2:1 and the adjusted parasite ratio is 1:2.4:0.6). Following tagmentation, the reaction was neutralized with NT buffer for 5 min and then the PCR Mastermix, consisting of Nextera PCR Master Mix, N700 index oligo, and N500 index oligo, was added to the reaction. PCR amplification was performed using the following cycling conditions: initial denaturation of 95°C for 30 s; 12 cycles of 95°C for 10 s, 55°C for 30 s, and 72°C for 30 s; and a final extension of 72°C for 5 min. The PCR products were isolated through two rounds of SPRI purification (0.6 × and 0.7 × volumetric ratios), and then assessed using an Agilent 4200 TapeStation instrument. Using the results from the TapeStation, individual libraries were normalized and pooled for sequencing.

Sequencing and FASTQ generation were performed by the Broad Genomics Platform. Samples were sequenced on two NovaSeq 6000 S2 flow cells using the following structure: 20 (Read 1), 50 (Read 2), 8 (Index 1), 8 (Index 2). Pre-processing, alignment to the ToxoDB v.41 assembly of the ME49 genome, and digital gene expression (DGE) generation was performed using V1 of the DropSeq pipeline (Macosko et al., 2015; http://mccarrolllab.org/dropseq/). To determine the number of parasites captured, an estimate of the number of single parasites was made using plotCumulativeFractionOfReads() from Dropbead in R with a maximum of 12,000 cells (Alles et al., 2017). The corresponding cells were then further parsed and analyzed using the Seurat R package (v. 2.3.4) (Butler et al., 2018).

In our analysis of all time points, genotypes and growth conditions (Figures 4E–4G), we required cells to contain a minimum of 200 and a maximum of 10,000 non-rRNA mapping unique molecular identifiers (UMIs) and have 40% or fewer total UMIs originating from rRNA. In our analysis of the 72 h time point (Figures 4A, 4B, 4H, and 4I), we required cells to contain a minimum of 500 and a maximum of 5,000 non-rRNA mapping UMIs, and contain 10% or fewer total UMIs originating from rRNA. These more stringent parameters result from higher confidence at this time point of what is and is not a cell, based on the tighter distribution of rRNA content and UMIs, with an upper limit of UMIs corresponding approximately to the mean plus 2 standard deviations.

Variable genes were identified through outlier analysis of an average expression by dispersion scatterplot using FindVariableGenes() and the following parameters: mean.function = ExpMean, dispersion.function = LogVMR, x.low.cutoff = 0.0125, y.cutoff = 0.5. Cells were log-normalized and scaled to 10,000 UMIs, regressing out the number of UMIs detected. Principal component analysis (PCA) was run using these variable genes. The number of principal components (PCs) chosen to use for clustering and UMAP visualization was based on permutation analysis and visual inspection of standard deviations of PCs.

For clustering of the 72 h time point for unstressed parasites only (Figures 4A and 4B), principal components 1–6 were used based on their identification as statistically significant (*p-value* < 0.001) by permutation as implemented by Seurat in JackStraw(). These PCs agreed well with visual inspection of the elbow seen when plotting the standard deviation of principal components. Clustering was done using shared nearest neighbor (SNN) as implemented by Seurat in FindClusters() using default parameters and a resolution of 0.6. Visualization by UMAP was done using a min_dist parameter of 0.3. For clustering of all parasites, time points and genotypes (Figures 4E and 4F), principal components 1–10 were selected for use, based on the elbow seen when plotting the standard deviation of principal components. Clustering was done as above using default parameters and a resolution of 0.8, and visualization by UMAP used min_dist = 0.5. For clustering of 72 h time point unstressed and stressed parasites (Figures 4H and 4I), principal components 1–18 were used based on their statistical significance by JackStraw(), and agreement by elbow plot analysis. Clustering was done as above using default parameters and a resolution of 0.8, and visualization by UMAP used min_dist = 0.5.

Differential gene expression between clusters or groups of clusters was performed using the Wilcoxon rank sum test as implemented by Seurat in FindMarkers(), using the following parameters: only.pos = TRUE, logfc.threshold = 0.5. For integration of the synchronized tachyzoite cell-cycle microarray data, raw microarray values were scaled and mean-centered per probe. For each cluster (Figure 4A), probes corresponding to differentially expressed genes were subset from the scaled and centered transformation, and averaged for each time point. Cluster 4 did not contain any differentially expressed genes and was therefore excluded from analysis. G1, S/M, and bradyzoite-specific gene signatures were derived from existing literature and from the stage-specific RNA-sequencing performed in this study (Data S1; Data S6) (Behnke et al., 2008, Fritz et al., 2012, Ramakrishnan et al., 2019, Suvorova et al., 2013). Cell scores calculated in Seurat using AddModuleScore() with default parameters.

#### RNA-seq and analysis of conditional BFD1 expression

WT (ME49Δ*KU80*), Δ*BFD1,* and Δ*BFD1/DD-BFD1-Ty* parasites were inoculated into T-12.5 flasks seeded with HFFs and allowed to invade for 4 h before exchanging media to standard media supplemented with either 3 μM Shield-1 or an equivalent amount of vehicle (100% ethanol). Parasites were grown for an additional 48 h before harvesting. Media was aspirated and monolayers rinsed with PBS. Following addition of 3 mL PBS, parasites were mechanically released by scraping the host cell monolayers and serially passing through 27- and 30-gauge needles, and filtering out debris through a 5 μm membrane. Parasite suspensions were centrifuged for 5 min at 1,000 × *g*, and PBS was aspirated, Pellets were resuspended in 1 mL TRIzol (Ambion), vortexed, and incubated at room temperature for 5 min before transferring to phase lock heavy tubes (Quantabio). 200 μL of chloroform was added to each tube, and the tubes were shaken vigorously for 15 s, followed by centrifugation at 12,000 × *g* for 15 min. The aqueous (top) layer was transferred to a fresh tube, and 1 μL glycoblue (Thermo Fisher) and 500 μL isopropanol added, followed by precipitation overnight at –20°C. The next day, samples were centrifuged at 12,000 × *g* for 5 min, washed twice with 75% ethanol, air-dried for 5 min, and resuspended in 30 μL RNase-free water. Samples were treated with TURBO DNase (Thermo Fisher) according to the manufacturer’s protocol, and RNA quality was assessed using a BioAnalyzer or Fragment analyzer. Library preparation was performed using the NEBNext Ultra II Directional RNA kit (NEB) following the polyA mRNA workflow. Paired-end sequencing (75/75 bp) was performed on an Illumina NextSeq. Reads were aligned against version 36 of the ME49 genome from ToxoDB using STAR (v 2.6.1) with–quantMode GeneCounts, as performed for our stage-specific RNA-seq. To allow for comparison with our stage-specific RNA-seq, which was prepared using an unstranded mRNA sequencing kit, reads were treated as unstranded as well. We note that the strandedness of our conditional BFD1 expression data allows for examination and assignment of reads to additional genes with overlapping annotations that cannot be distinguished using unstranded data, though this precludes comparison to our alkaline stress regulation data.

#### CUT&RUN

##### pAG-MNase purification

The His-tagged pAG-MNase fusion protein construct generated by Meers et al. was generously provided by Mary Gehring, while the protein purification protocol was derived and modified from Meers et al. (Meers et al., 2019). The purified DNA construct was first transformed into chemical competent JM101 cells (Agilent) according to the manufacturer’s instructions and grown in NZYM-Kanamycin medium (50 μg/mL). At an OD_600_ of 0.55, expression of the pAG-MNase protein was induced for 2 h at 37°C by the addition of Isopropyl-β-D-1-thiogalactopyranoside (Thermo Fisher) to a final concentration of 2 mM. Cells were harvested by centrifugation at 3,500 × *g* for 20 min, washed with ice cold PBS and lysed by resuspension of the pellet in 10 mL lysis buffer (10 mM Tris-HCl pH 7.5, 300 mM NaCl, 10 mM Imidazole, 5 mM β-Mercaptoethanol) containing 10 μg/mL chicken egg white lysozyme (Sigma-Aldrich). Following 15 min incubation at 30°C, cells were sonicated on ice using a Branson Sonifier 250 (VWR) at output control level 4 for 12 intervals of 45 s. The lysate was cleared by centrifugation at 12,000 × *g* for 20 min at 4°C and loaded onto a Ni-NTA Agarose column (QIAGEN) previously washed twice with two bed volumes of lysis buffer. To bind the His-tagged pAG-MNase fusion protein to the beads, the suspension was incubated on a rotator for 1 h at 4°C before loading on a disposable gravity column (Bio-Rad) and washing twice with 5 bed volumes of wash buffer (10 mM Tris-HCl pH 7.5, 300 mM NaCl, 20 mM Imidazole, 0.03% Zwittergent 3-10 Detergent). After elution of the pAG-MNase in 1 mL elution buffer (10 mM Tris-HCl pH 7.5, 300 mM NaCl, 250 mM Imidazole), the eluate was dialyzed twice at 4°C using a Slide-A-Lyzer Dialysis Cassette (Thermo Fisher) against 750 mL dialysis buffer (10 mM Tris-HCl pH 7.5, 150 mM NaCl, 1 mM EDTA, 1 mM PMSF) for 2 h and overnight, respectively. Aliquots were stored after addition of glycerol to a final concentration of 50% at –20°C.

##### CUT&RUN

Δ*BFD1*/*DD-BFD1*-Ty parasites were maintained in standard medium. For bradyzoite conversion, the medium was changed 24 h post-infection to alkaline stress medium (pH 8.1). To remove any residue of the previous medium, cells were washed once with alkaline stress medium before incubation. Bradyzoites were maintained for 48 h at 37°C at ambient CO_2_ and harvested by mechanically releasing by serially passing through 30- and 27-gauge syringes before filtering through a 5 μm membrane. Parasites were then processed according to a modified version from Skene and Henikoff as follows (Skene and Henikoff, 2017). 1 × 10^7^ filtered bradyzoites were pelleted by centrifugation at 600 × *g* for 10 min and washed in 1.5 mL wash Buffer 1 (20 mM HEPES-NaOH pH 7.5, 150 mM NaCl, 0.5 mM Spermidine (Sigma-Aldrich) and a cOmplete Mini EDTA-free Protease Inhibitor Cocktail tablet (Roche Diagnostics) per 10 ml). Parasite nuclei were isolated by resuspension of the washed parasite pellet in Lysis Buffer (10 mM HEPES-KOH pH 7.9, 1.5 mM MgCl_2_, 10 mM KCl, 0.65% IGEPAL CA-630 (Sigma-Aldrich) and a cOmplete Mini EDTA-free Protease Inhibitor Cocktail tablet per 10 mL). Parasites were lysed on ice for 10 min and centrifuged at 1500 × *g* for 10 min at 4°C to isolate the nuclear fraction. Nuclei were resuspended in 50 μL Binding Buffer (20 mM HEPES-KOH pH 7.9, 10 mM KCl, 1 mM CaCl_2_, 1 mM MnCl_2_, 0.5 mM Spermidine) and bound to 25 μL BioMag Plus Concanavalin A coated magnetic beads (Polysciences) by rotating at low speed for 10 min at room temperature. BioMag Plus Concanavalin A coated magnetic beads were previously activated by washing twice with 100 μL Binding Buffer while the sample tube was centrifuged at 500 × *g* for 3 s to remove any remaining liquid from the lid after each rotation step. Using a magnetic rack, the remaining binding buffer was decanted and nuclei were blocked in 500 μL Blocking Buffer (20 mM HEPES-NaOH pH 7.5, 150 mM NaCl, 0.5 mM Spermidine, 2 mM EDTA and 0.1% BSA (Sigma-Aldrich)) by inverting 10 times and incubation for 5 min at room temperature. After 5 min, the remaining blocking buffer was decanted using a magnetic rack and the sample was washed with 350 μL Wash Buffer 2 (20 mM HEPES-NaOH pH 7.5, 150 mM NaCl, 0.5 mM Spermidine, 0.1% BSA and a cOmplete, Mini, EDTA-free Protease Inhibitor Cocktail tablet per 10 mL). Thereupon, 200 μL of the 1:1,000 diluted anti-Ty antibody (BB2) or mouse IgG isotype control (Thermo Fisher) in Wash Buffer 2 were added to the sample and incubated with gentle rotation at 4°C for 2 h. The remaining antibody was washed off using two volumes of 350 μL Wash Buffer 2 and inverting 10 times, followed by the addition of 0.1 μg pAG-MNase in 200 μL Wash Buffer 2. To allow the pAG-MNase to bind to the anti-Ty or isotype control antibody, the sample was incubated at 4°C for 1 h with gentle rotation. The remaining pAG-MNase was removed by washing the beads twice with 350 μL Wash Buffer 2 and inverting 10 times. Prior the cutting reaction, the sample was resuspended in 100 μL Wash Buffer 2 and equilibrated to ∼0°C for 5 min on a metal block in wet ice. The cutting reaction was then started by spiking in CaCl_2_ to a final concentration of 2 mM and incubating for 30 min at 0°C before quenching by addition of 100 μL 2 × Stop Buffer (200 mM NaCl, 20 mM EDTA, 4 mM EGTA, 50 μg/mL RNase A (Thermo Fisher) and 40 μg/mL glycogen (Thermo Fisher)). To release the CUT&RUN fragments into the supernatant and digest RNA, the sample was incubated at 37°C for 20 min and fractionated on a magnetic rack.

To extract CUT&RUN fragments from solution, an organic extraction and ethanol precipitation modified from Zheng and Gehring was performed (Zheng and Gehring, 2019). After fractionation of the CUT&RUN fragments, SDS and proteinase K (Life Technologies) were added to final concentrations of 0.1% and 0.2 mg/mL, respectively, and incubated for 10 min at 70°C. To extract DNA, 300 μL buffered Phenol:Chloroform:Isoamyl solution (Invitrogen) were added and then vortexed at low speed. Phases were separated using Phase Lock Gel Light tubes (Quantabio) and centrifugation at 21,000 × *g* for 5 min, followed by the addition of 300 μL chloroform and centrifugation. The aqueous phase containing the DNA fragments was transferred to a fresh tube containing 2 μL of 2 mg/mL glycogen and DNA was precipitated by the addition of 750 μL ice cold 100% ethanol and vortexing. After the suspension was incubated on ice for 10 min, the DNA was pelleted by centrifugation at 21,000 × *g* for 15 min. The ethanol was decanted and the DNA pellet dried for 5 min before it was washed with 1 mL 100% ethanol, centrifuged at 21,000 × *g* for 10 min and dried again. Finally, the dry pellet was dissolved in 38 μL of 0.1 × TE8 (1 mM Tris, 0.1 mM EDTA pH 8).

##### Library preparation

CUT&RUN libraries were prepared using a protocol modified from Zheng and Gehring (Zheng and Gehring, 2019). Purified CUT&RUN fragments were first end-repaired, phosphorylated and A-tailed by adding 36.5 μL of CUT&RUN fragment solution to 13.5 μL of 3.7 × ERA buffer containing 3.7 × T4 DNA ligase Buffer (NEB), 2 mM dNTPs (Kapa Biosystems), 1 mM ATP (NEB), 9% PEG4000 (EMD Millipore), 0.5 U/μL T4 Polynucleotide Kinase (NEB), 0.019 U/μL T4 DNA Polymerase (NEB), 0.019 U/μL Taq DNA Polymerase (Thermo Fisher). The following program was used on a pre-cooled thermal cycler: 12°C for 15 min, 37°C for 15 min, and 72°C for 20 min. Immediately after A-tailing, adaptor ligation was performed by adding 5 μL of TruSeq DNA Single Indexes Set A (Illumina) diluted 1:50 in water, and 60 μL quick ligase solution containing 2 × Quick Ligase Reaction Buffer and 50 U/μL Quick Ligase (Enzymatics) to the A-tailed library and incubating for 15 min at 20°C on a pre-cooled thermal cycler without a heated lid. Immediately after adaptor ligation, ligated DNA fragments were purified through two rounds of RNAClean XP bead cleanup. In the first round, 38 μL (0.33 × ) RNAClean XP bead solution (Beckman Coulter) were added to the ligated DNA and incubated for 15 min to allow the DNA to precipitate onto the beads. The supernatant was decanted using a magnetic rack, the beads were washed twice with 200 μL 80% ethanol and air-dried for 5 min at room temperature. DNA was eluted from RNAClean XP beads using 53 μL of 10 mM Tris-HCl pH 8. In the second round, 50 μL of eluted DNA was added to 55 μL (1.1 × ) RNAClean XP bead solution, precipitated, and washed similarly to the first round, but eluted in 22 μL of 10 mM Tris-HCl pH 8. Library enrichment was performed by adding 30 μL of KAPA PCR Master Mix containing 1.6 × KAPA HiFi HotStart ReadyMix (Kapa Biosystems), 0.3 μM PAGE purified P5 (IDT, 5′-AATGATACGGCGACCACCGA^∗^G) and 0.3 μM PAGE purified P7 (IDT, 5′-CAAGCAGAAGACGGCATACGA^∗^G) to 20 μL of cleaned, re-eluted DNA fragments. The following cycling conditions were used for library enrichment: 98°C for 45 s; 13 cycles of 98°C for 15 s, and 60°C for 10 s; and 72°C for 1 min. The enriched library was purified through two rounds of RNAClean XP bead cleanup as described above. For both rounds 55 μL (1.1 × ) RNAClean XP bead solution was used and the enriched library was eluted with 52 μL dH_2_O after the first round and 18 μL of dH_2_O after the second round of cleanup. Sequencing of pooled libraries was performed using 25 bp × 25 bp paired-end sequencing and a MiSeq Reagent Kit v2 (Illumina).

##### Data processing and analysis

Paired-end sequencing reads were trimmed and filtered using trim_galore (version 0.4.1) with a quality cutoff score of 20, a stringency of 3 and a minimal sequence length of 10. The resulting reads were aligned to the ME49 genome (https://www.toxodb.org, release 45) using bowtie2 (version 2.3.4.1) (Langmead and Salzberg, 2012) with the following options:

–local–very-sensitive-local–no-unal–no-mixed–no-discordant–phred33 -I 10 -X 700

Peak calling was performed using MACS2 (version 2.1.1.20160309) with the following options:

g 65668394–nomodel -B –extsize 183

The estimated fragment length was calculated by strand cross-correlation analysis using R version 3.6.1 on RStudio version 1.1.463 (RStudio, Inc.) with the package ChIPQC (version 1.20.0).

Motif analysis using MEME (version 4.11.4) was performed on peaks that were called on DNA fragments ≤ 120 bp to increase the sequence proportion containing the motif using the settings:

dna -nmotifs 10 -minw 4 -maxw 14 –revcomp

Motif analysis using HOMER (version 4.10) was performed on similar DNA fragments as for MEME using the settings:

findMotifs.pl -len 8,9,10,11,12

Sequences for motif analysis were extracted from the ME49 genome using R and RStudio using the packages tidyr (version 1.0.0) and ChIPQC. Strand cross-correlation was used to determine the sequence size excerpted, using the called peak position as the midpoint. To remove low complexity sequences, a slow search on RepeatMasker (version 4.0.7) with species setting apicomplexan was performed. The background file for MEME and HOMER was generated with the function fasta-get-markov as implemented in MEME, using the median peak length of called peaks from genes as the distance from the transcription start sites (TSS) of genes with no assigned peaks.

MEME motif occurrences in the whole ME49 genome were identified using FIMO with a threshold of *p-value* < 0.0001. Fragments ranging from 443 bp upstream to 494 bp downstream of all transcriptional start sites were scanned, which represents the regions in which 50% of BFD1 peaks were identified. As background sequences, regions upstream of the 5% quantile of equal length were used. Motif occurrences within BFD1 peaks called using fragments ≤ 700 bp were identified by scanning the full length peak fragment with a *p-value* threshold < 0.0001 and with a background file of 0-order letter frequencies contained in the motif file.

Gene IDs were assigned to peaks based on the distance of each peak to the closest TSS, taking into account the direction in which the gene is read, using the packages tidyverse (version 1.2.1), plyr (version 1.8.4), data.table (version 1.12.2), dplyr (version 0.8.3), DescTools (version 0.99.28), ape (version 5.3) and tidyr.

MACS2 identified 815 peaks, which were filtered by removing peaks from chromosomes without genes (13 peaks), peaks that were assigned to multiple genes (76 peaks), and genes with a peak distance outside of the 5–95% quantiles of all distances (74 peaks). Peaks were considered assigned to multiple genes if the peak-to-gene distance difference between the closest gene and the second closest gene to the peak lies below the 75% quantile of the peak-to-gene distance of the closest assigned gene (1068 bp). Subsequently, heatmaps were generated with the remaining 652 peaks, using the R packages genomation (version 1.16.0) EnrichedHeatmap (version 1.14.0), ComplexHeatmap (version 2.0.0) and GenomicRanges (version 1.36.1). Genes were assigned to one of the following three categories based on the bulk stage-specific RNA-sequencing data: Significantly upregulated in bradyzoites (adjusted *p-value* < 0.001, log_2_ fold-change > 0) represented by “up in stress,” significantly downregulated in bradyzoites (adjusted *p-value* < 0.001, log_2_ fold-change < 0) represented by “down in stress,” and those lacking a significant change (adjusted *p-value* > 0.001 or unrepresented) represented by the category “unregulated.” Violin plots were generated using ggplot2 (version 3.2.1).

### Quantification and Statistical Analysis

Information including number biological replicates, number of observations made, and exact statistical tests used can be found in the relevant figure legends. For gene disruption, differentiation assessment in cell culture, and brain cyst formation, statistical tests were performed in Prism (GraphPad), and *p-value*s less than 0.05 were considered significant. For stage-specific bulk RNA-sequencing and conditional overexpression of *BFD1* RNA-sequencing, adjusted *p-value*s as calculated by DESeq2 were used (Love et al., 2014). For single-cell RNA-sequencing, adjusted *p-value*s as calculated by Seurat were used (Satija et al., 2015). Data was generated and analyzed without blinding, with the exception of brain cyst quantification as detailed in the methods.

### Data and Code Availability

https://www.ncbi.nlm.nih.gov/geo/query/acc.cgi?acc=GSE132250 The accession number for the data reported in this paper is GEO: GSE132250. This study did not generate any custom code. The analysis pipelines supporting the current study are available from the corresponding author on request.

## Supplemental Information

Data S1. Differential Gene Expression Analysis of Stage-Specific RNA Sequencing, Related to Figure 1 Log_2_-fold changes correspond to bradyzoites compared to tachyzoites, as shown in Figure 1D.

Data S2. Data from Libraries Used in Cas9-Mediated Screening for Differentiation Mutants, Related to Figure 1 Both raw gRNA counts and analysis at the gRNA and gene level are provided.

Data S3. Differential Gene Expression Analysis of Single-Cell RNA Sequencing, Related to Figure 4 For differentially expressed markers identified for clusters corresponding to the tachyzoite cell cycle, cluster names and cells used correspond to Figure 4A, and log-fold changes correspond to cells in the named cluster compared to cells in all other clusters. For differentially expressed genes identified between tachyzoite and bradyzoite-containing clusters, tachyzoite clusters (4, 5, 6, 8 and 9), bradyzoite clusters (0, 1, 7, 11 and 12), and cells used correspond to Figure 4H. Log-fold changes correspond to bradyzoite clusters compared to tachyzoite clusters. For markers identified for clusters corresponding to tachyzoites, bradyzoites, and stressed Δ*BFD1* parasites, cluster names and cells used correspond to Figure 4H, and log-fold changes correspond to cells in the named cluster compared to cells in all other clusters. For genes identified as differentially expressed between unstressed and stressed wild-type or Δ*BFD1* parasites split by time point, log-transformed fold changes correspond to alkaline-stressed parasites compared to unstressed parasites.

Data S4. Differential Gene Expression Analysis of a Conditionally Stabilized BFD1 Expression Strain, Related to Figure 5 Log_2_-fold changes correspond to Shield-1-treated ΔBFD1/DD-BFD1-Ty parasites compared to vehicle-treated parasites, as shown in Figure 5I.

Data S5. BFD1 Peaks Identified through CUT&RUN, Related to Figure 6 Peak locations, filtering criteria, and gene assignments as shown in Figure 6B.

Data S6. Primers, Plasmids, and Gene Modules Used in This Study, Related to STAR Methods
